# Supplementary figures and images for: Deep mutational scanning of Pneumocystis jirovecii dihydrofolate reductase reveals allosteric mechanism of resistance to an antifolate
Source: PLoS Genet. 2024 Apr 29;20(4):e1011252. doi: 10.1371/journal.pgen.1011252 (PMC11125491; doi:10.1371/journal.pgen.1011252)

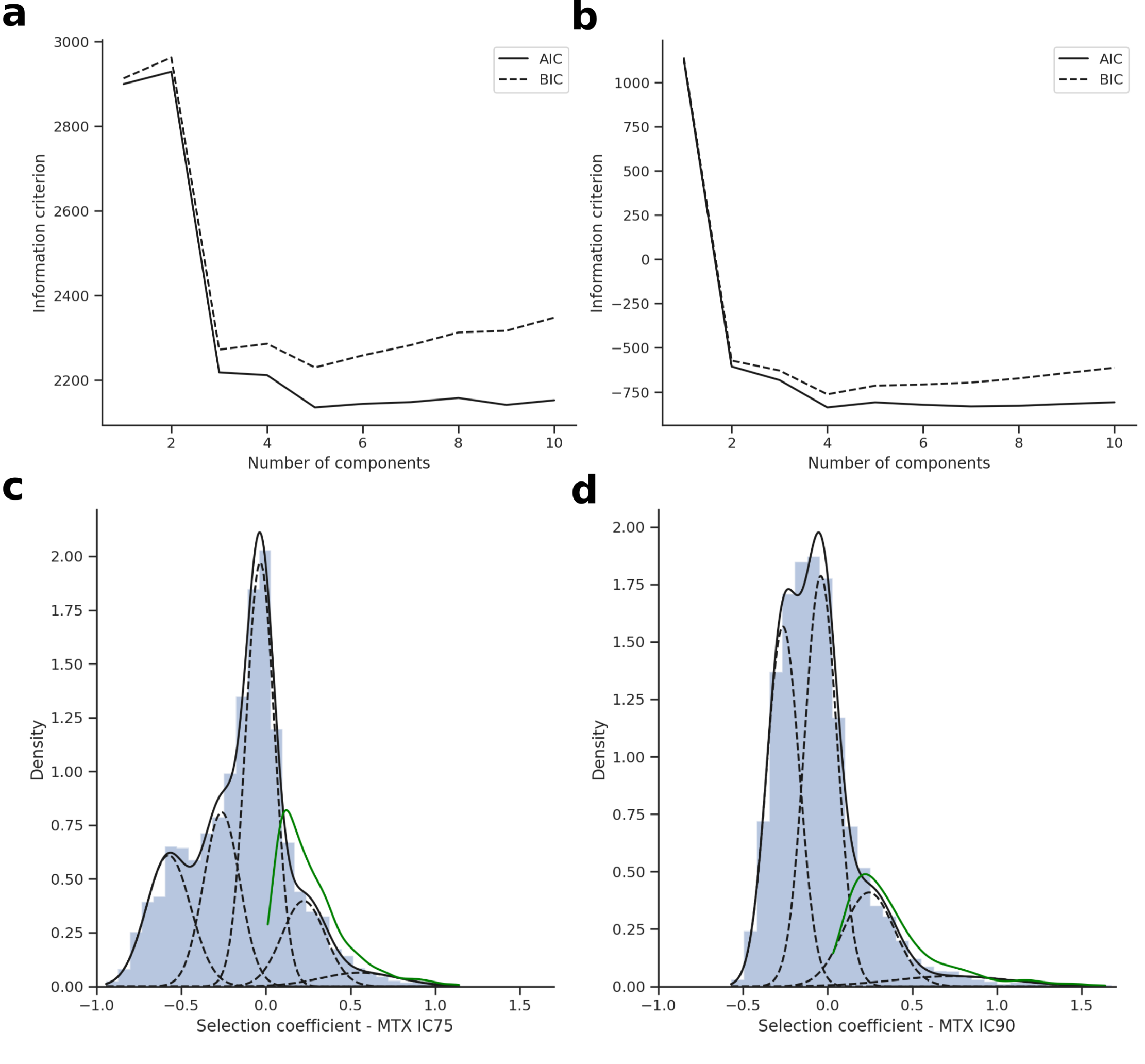

Supplement: S1 Fig — Optimization of information criterion for a) IC75 and b) IC90. Best Gaussian mixture model (dashed lines represent underlying Gaussians) to recapitulate the underlying distribution (black line/histogram) of c) IC75 and d) IC90. Density curve of significant mutants for Benjamini-Hochberg (green) correction is visible. (TIFF) [file pgen.1011252.s001.tiff]

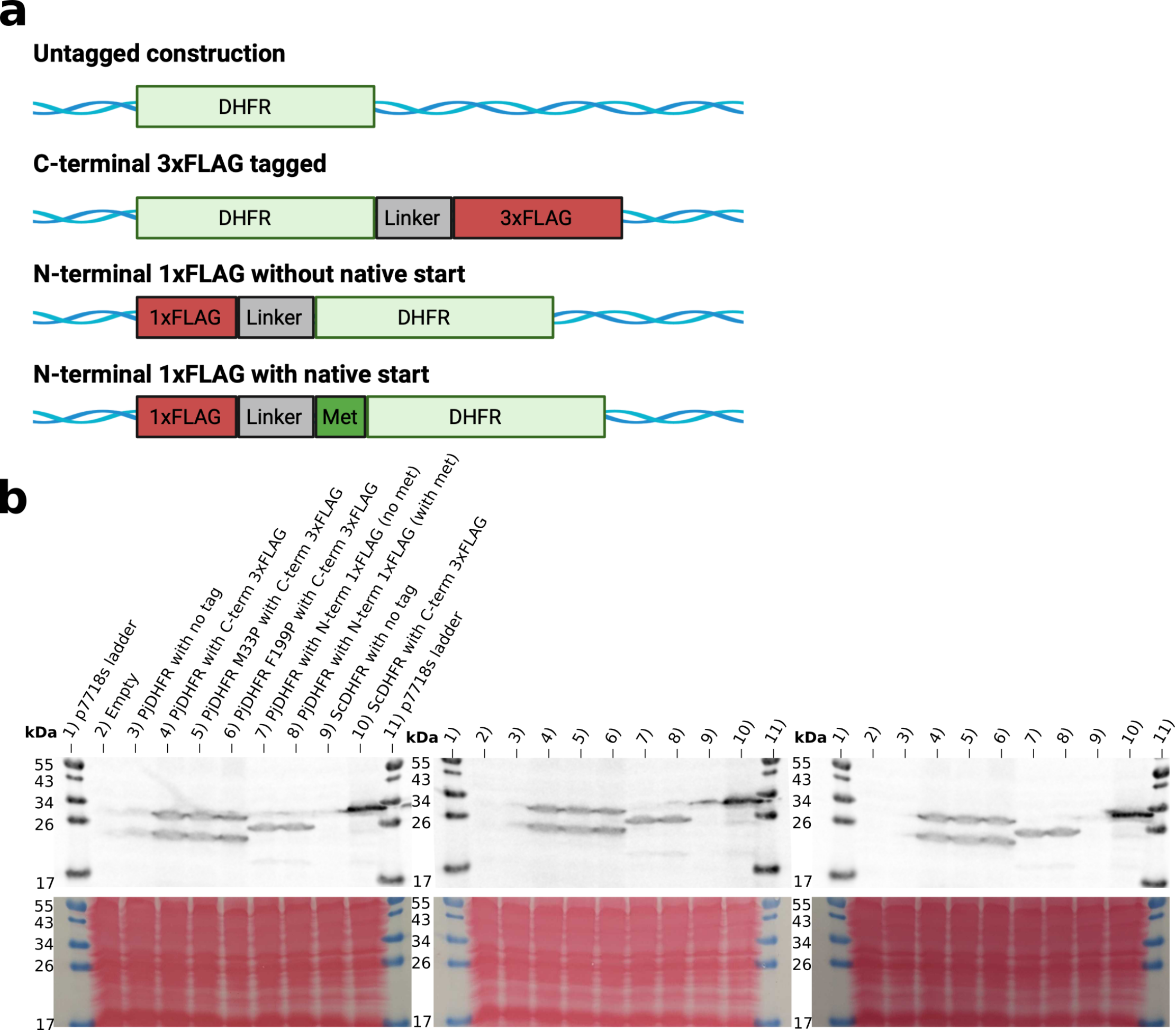

Supplement: S2 Fig — a) Construction schematics of tagged proteins expressed in yeast that can further be detected in protein cell extracts by western blots. All proteins tagged at their C-termini are tagged using 3xFLAG-tag, and all proteins tagged at their N-terminal are tagged using 1xFLAG-tag. N-terminal-tagged proteins were constructed with or without their native start codon. Constructions are not to scale. Schematic was made with BioRender. b) Western blots from biological replicates for all constructions. In C-terminal-tagged constructions with PjDHFR (wells 4-5-6), two bands can be seen, corresponding to the full length (top band) and the truncated protein from an alternative start codon (possibly M33 or M34, lower band). This lower band is absent from the wells with the N-terminal constructions (wells 7–8), supporting the hypothesis of an alternative start codon versus a post-translational cleavage. (TIFF) [file pgen.1011252.s002.tiff]

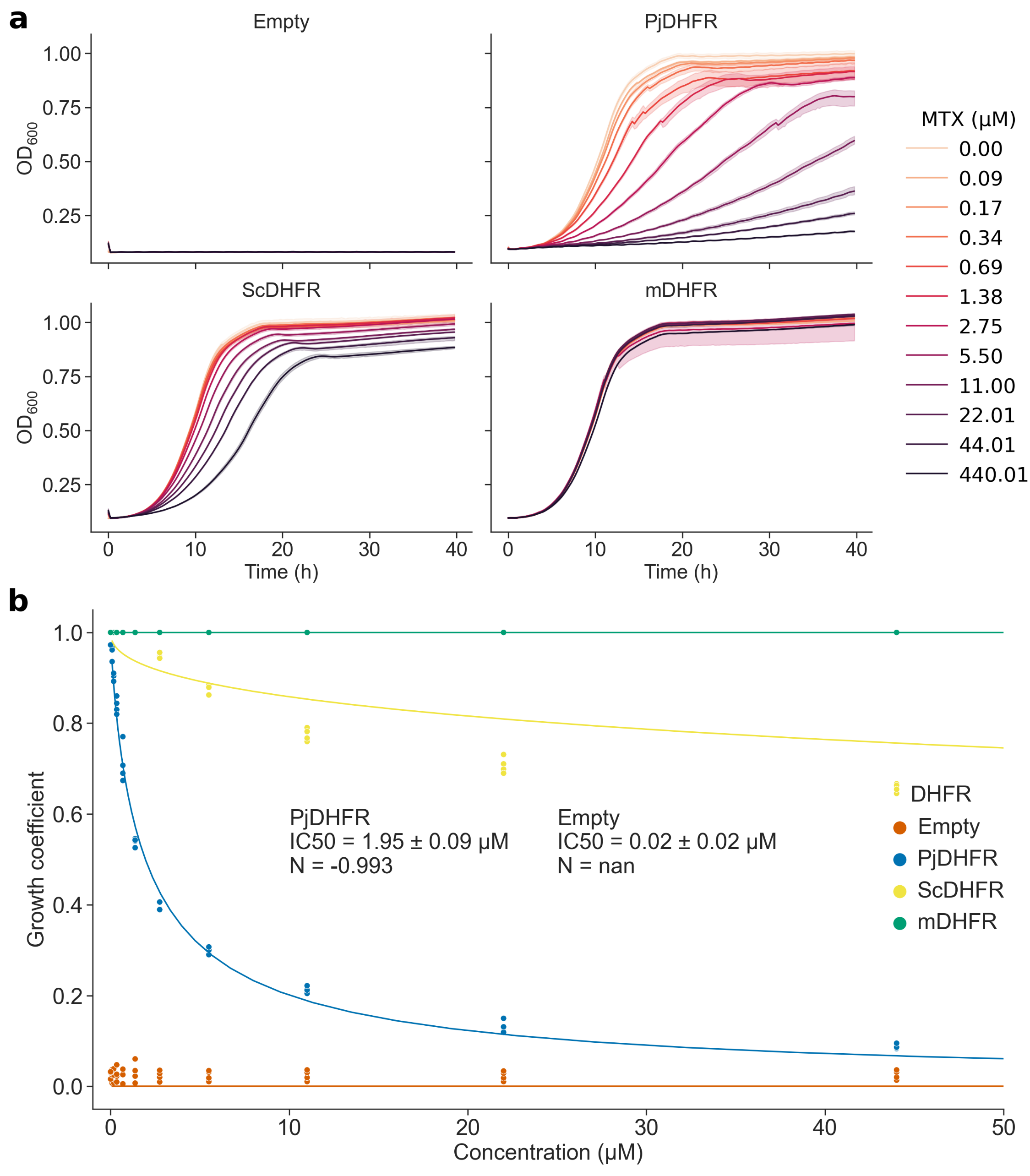

Supplement: S3 Fig — a) Growth curves of yeast strain FDR0001 (dfr1Δ) each expressing a different DHFR. Shaded areas represent confidence intervals across biological triplicates. The Empty vector does not support growth in this strain. Strains with vector-expressed DHFRs show increased resistance, with PjDHFR being the most sensitive, followed by ScDHFR, and mDHFR(L22F/F31S) being fully resistant. b) Growth coefficient of strain FDR0001 with different DHFRs. R2 for Hill equation fit: Empty = null, PjDHFR = 0.99, ScDHFR = 0.73 and mDHFR = >0.99. (TIFF) [file pgen.1011252.s003.tiff]

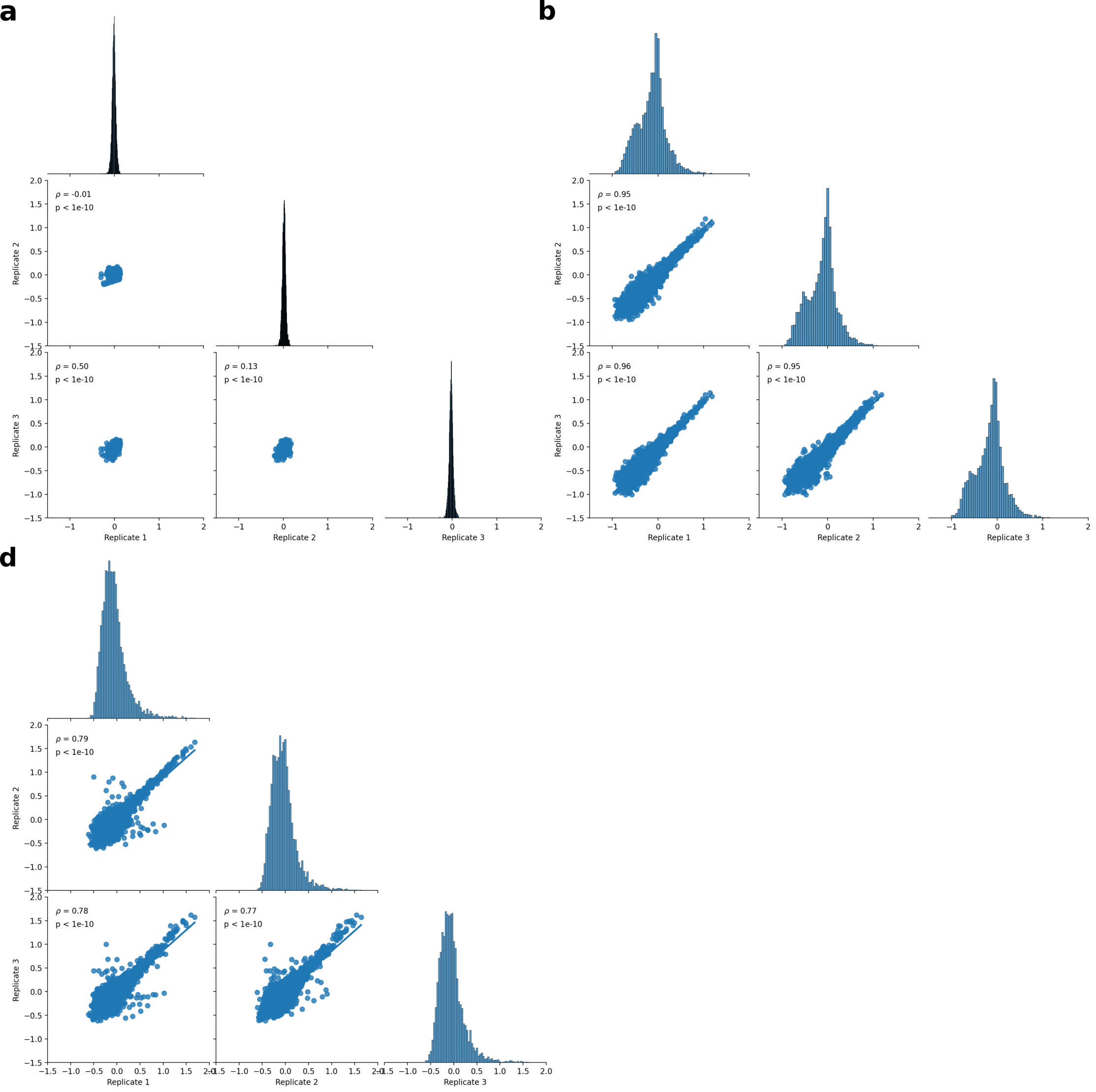

Supplement: S4 Fig — a) Distributions of selection coefficients for amino acids in the different replicates in DMSO condition. Axes are scaled to allow comparison of the different conditions. Since the distribution of selection coefficients in DMSO is much narrower than in MTX, data appears as a group centered on 0. Density plots show the distribution of selection coefficients for the replicate on the x-axis. b) Distributions of selection coefficients for amino acids in the different replicates in MTX IC75 condition. c) Distributions of selection coefficients for amino acids in the different replicates in MTX IC90 condition. Statistical tests are Spearman’s rank correlation. (TIFF) [file pgen.1011252.s004.tiff]

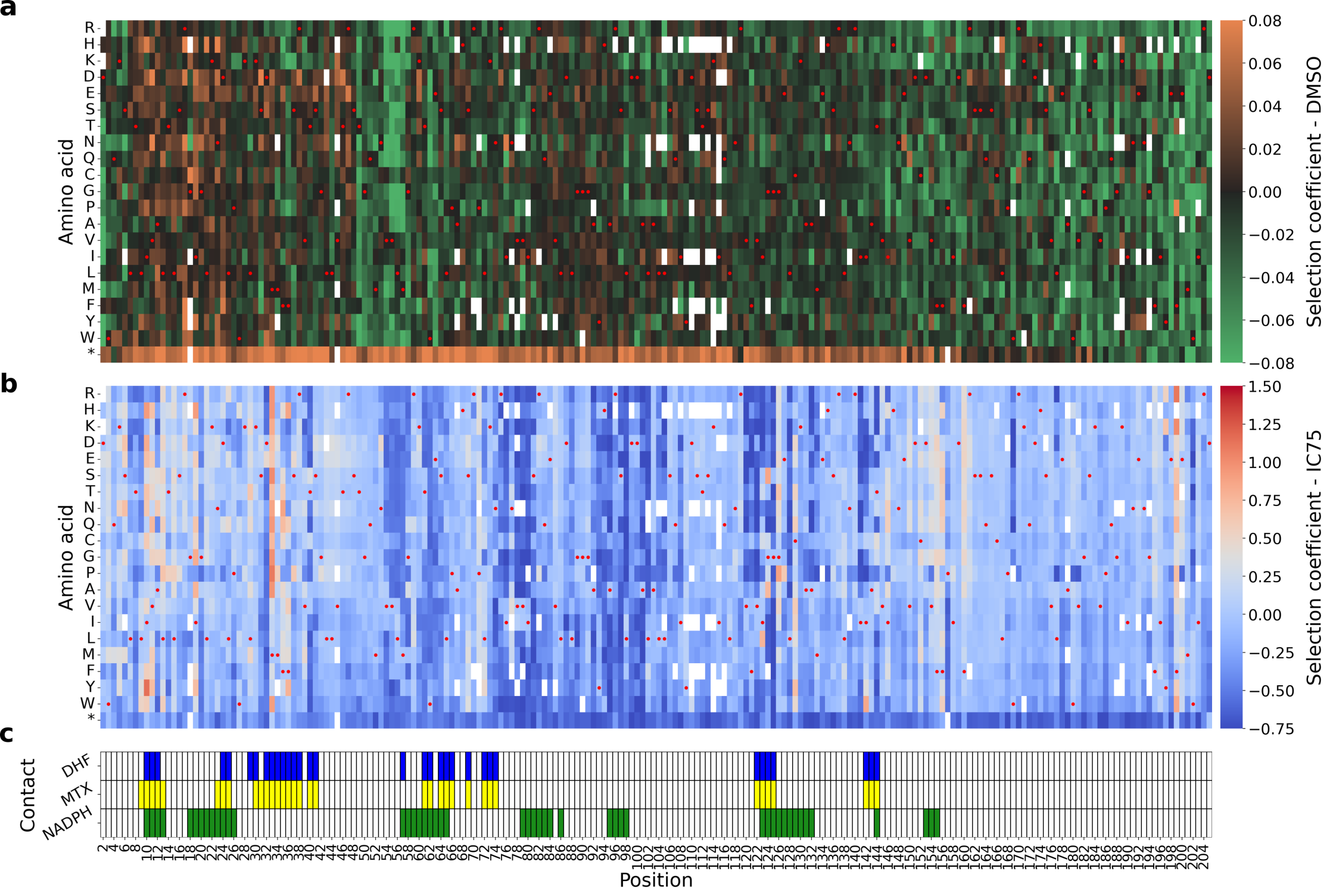

Supplement: S5 Fig — a) Selection coefficient of a given amino acid (y-axis) at a given position (x-axis) without MTX. Red points show the wild-type sequence of PjDHFR. b) Selection coefficient of a given amino acid (y-axis) at a given position (x-axis) at a MTX concentration corresponding to 75% of growth inhibition (IC75). c) Positions of contacting residues along PjDHFR modeled on structural alignments between PjDHFR and orthologous P. carinii DHFR (PDB: 3CD2 (MTX and NADPH) and 4CD2 (DHF)). Contact was established as amino acids with an α carbon located less than 8 Å from MTX, DHF or NADPH. (TIFF) [file pgen.1011252.s005.tiff]

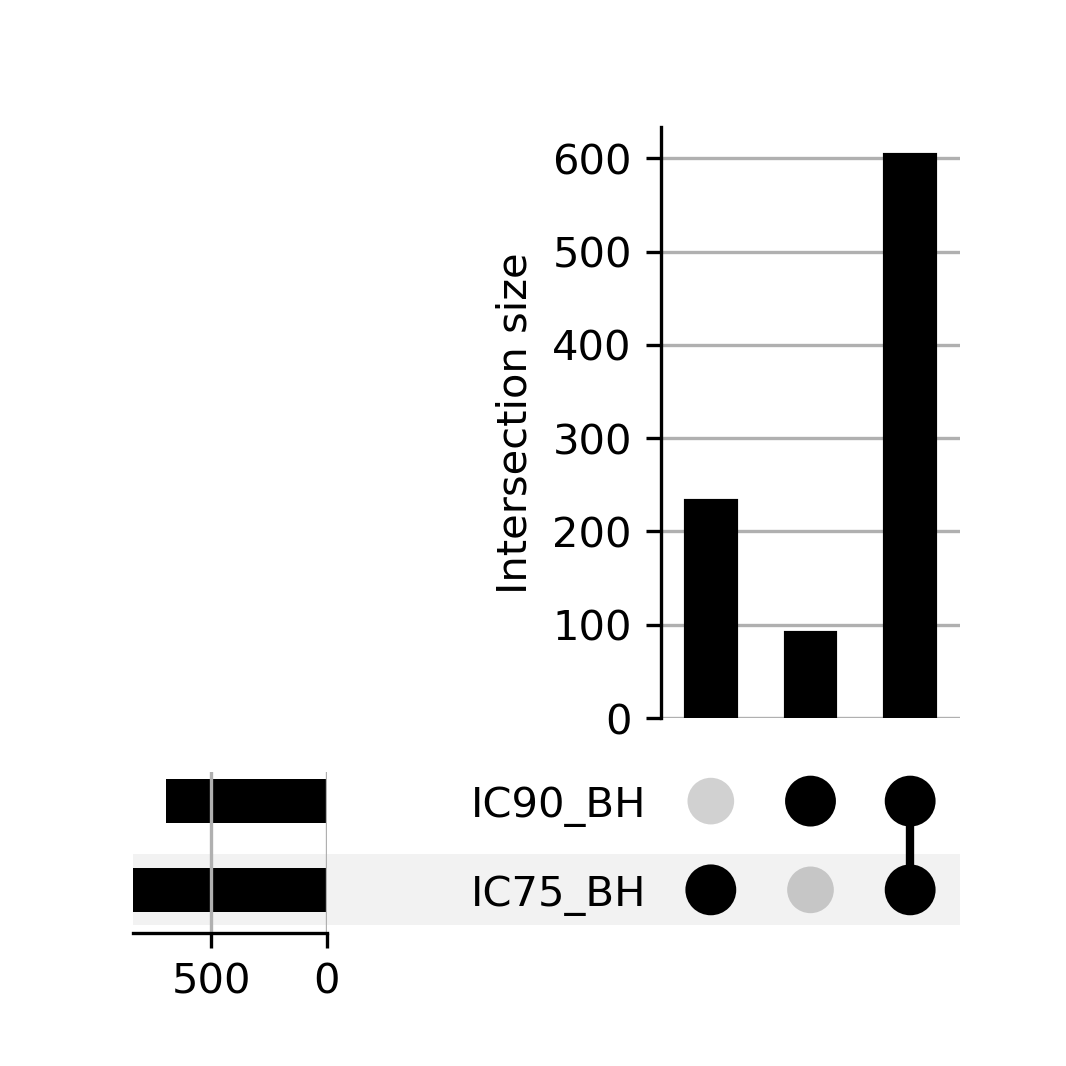

Supplement: S6 Fig — Upset plot showing the intersections of the Benjamini Hochberg-FDR corrected groups (control for the false discovery rate of significantly resistant mutants at 5% confidence). The x-axis bars represent the size of each group and the y-axis represent intersection size for each group. For maximum confidence, mutations considered significant in both conditions should be considered. (TIFF) [file pgen.1011252.s006.tiff]

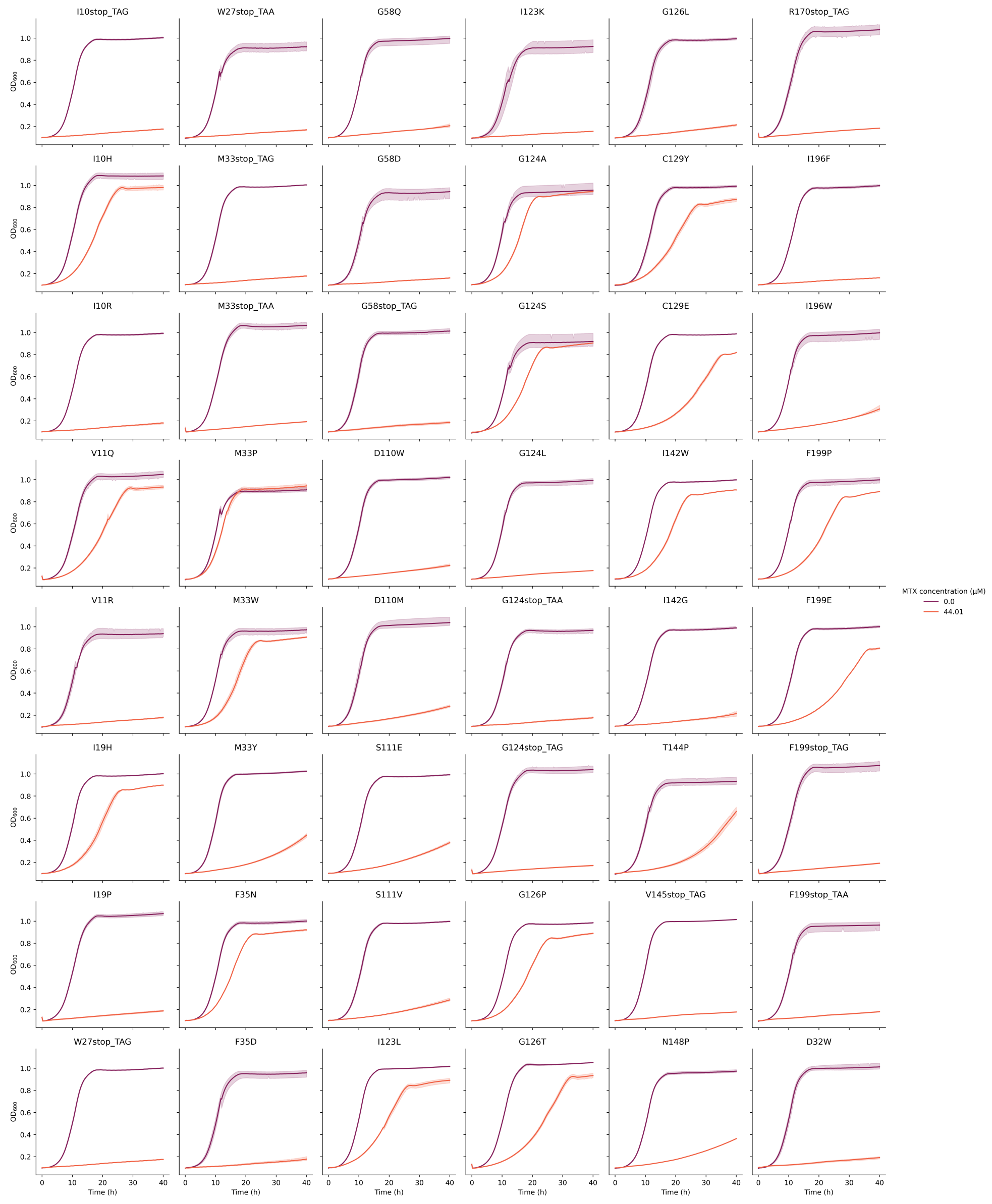

Supplement: S7 Fig — Mutants are identified on top of their individual growth curves. For stop codons, we constructed both TAG (from DMS using NNK degenerate primers) and TAA as extra controls to ensure that TAG stop codons did not allow read through. All curves were done in triplicate, with error intervals between replicates appearing around the curve in a lighter color. Strains were grown in the same media as the competition assay for 40 hours, and growth rates were measured from these curves. Small discrepancies intervals in some of the curves in DMSO can be attributed to the formation of bubbles within the wells of the plates. These intervals were not considered when measuring growth rate. (TIFF) [file pgen.1011252.s007.tiff]

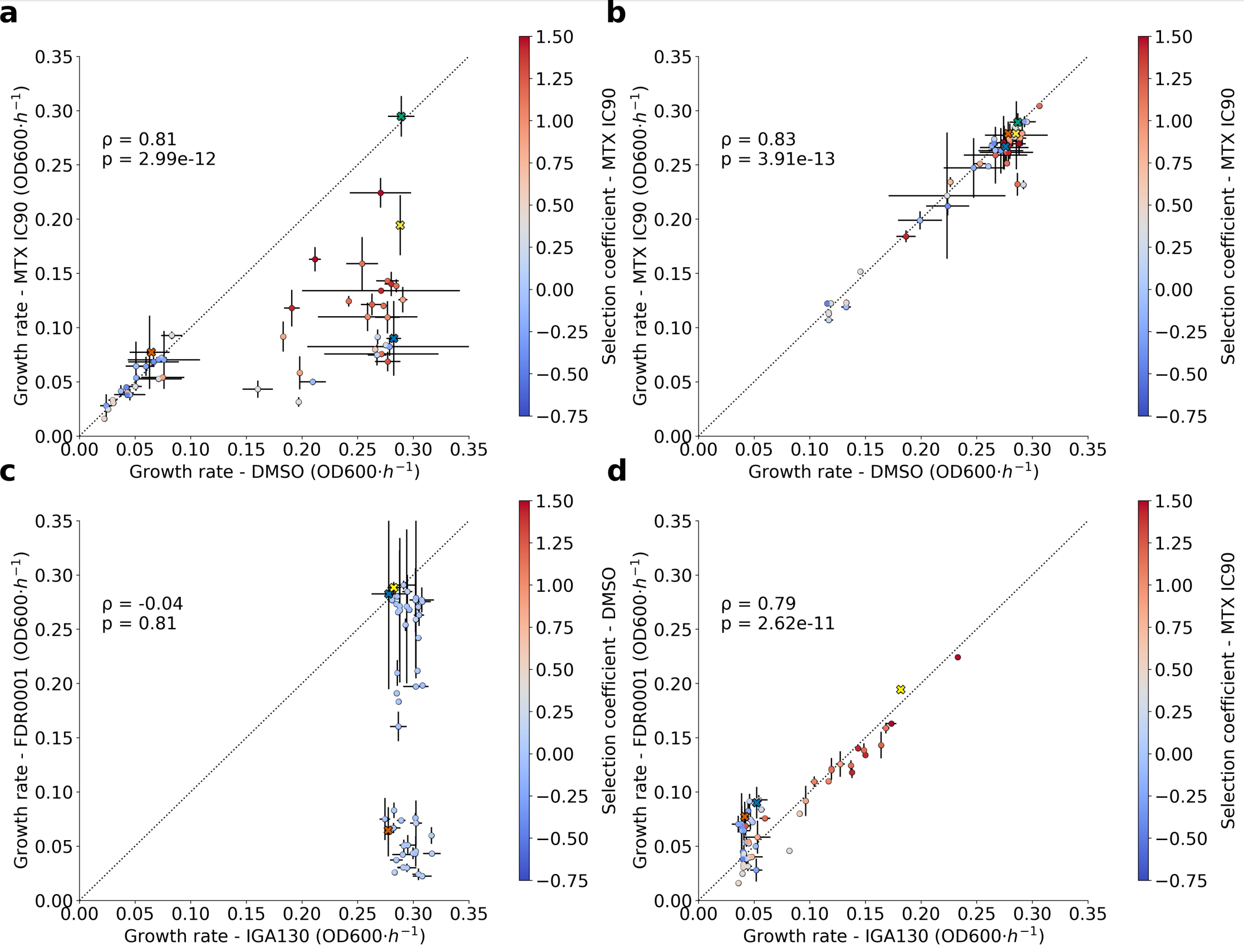

Supplement: S8 Fig — a) Growth rates in DMSO are plotted on the x-axis, as a measure of complementation, and growth rates in the presence of MTX IC90 are plotted on the y-axis, as a measure of resistance. These growth curves were conducted in the absence of β-estradiol. Many sensitive mutants cannot complement the deletion of DFR1 in strain FDR0001 (dfr1Δ). Error bars represent growth rate values measured across the biological replicates in both conditions. Data points marked by crosses represent the Empty plasmid (orange), the wild-type PjDHFR (blue), ScDHFR (yellow) and mDHFR (green). Other round data points are colored by their selection coefficients at IC90 unless specified otherwise. b) Same as panel a), but in the presence of β-estradiol, which leads to expression of MTX insensitive DfrB1. c) Comparison of growth rates between strains IGA130 (x-axis) and FDR0001 (y-axis) validation mutants in DMSO. All mutants can grow similarly in strain IGA130, but some mutants were non-functional in strain FDR0001. Color bar is the selection coefficient in DMSO. d) Comparison of growth rates between strains IGA130 (x-axis) and FDR0001 (y-axis) validation mutants in MTX IC90. For mutants that were identified as resistant, there is a strong correlation between growth rates measured in strain IGA130 and FDR0001, highlighting the minimal effect of the genomic DFR1 on MTX resistance. Non-functional mutants in FDR0001 had to be supplemented with 100 nM β-estradiol to ensure growth of precultures. Statistical tests are Spearman’s rank correlation. (TIFF) [file pgen.1011252.s008.tiff]

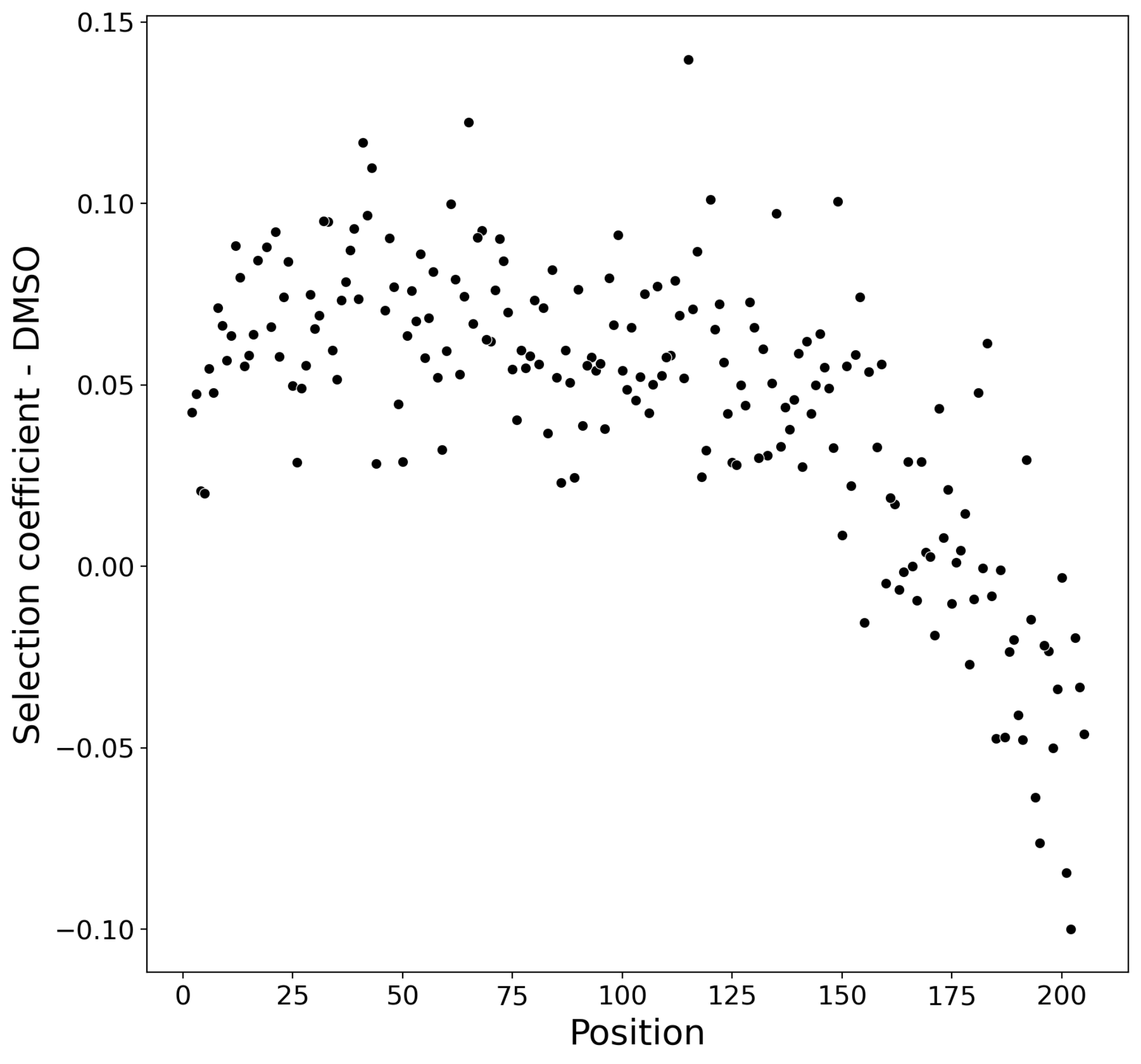

Supplement: S9 Fig — Each point represents the selection coefficient of a stop codon at a given position (the median selection coefficient across triplicates). (TIFF) [file pgen.1011252.s009.tiff]

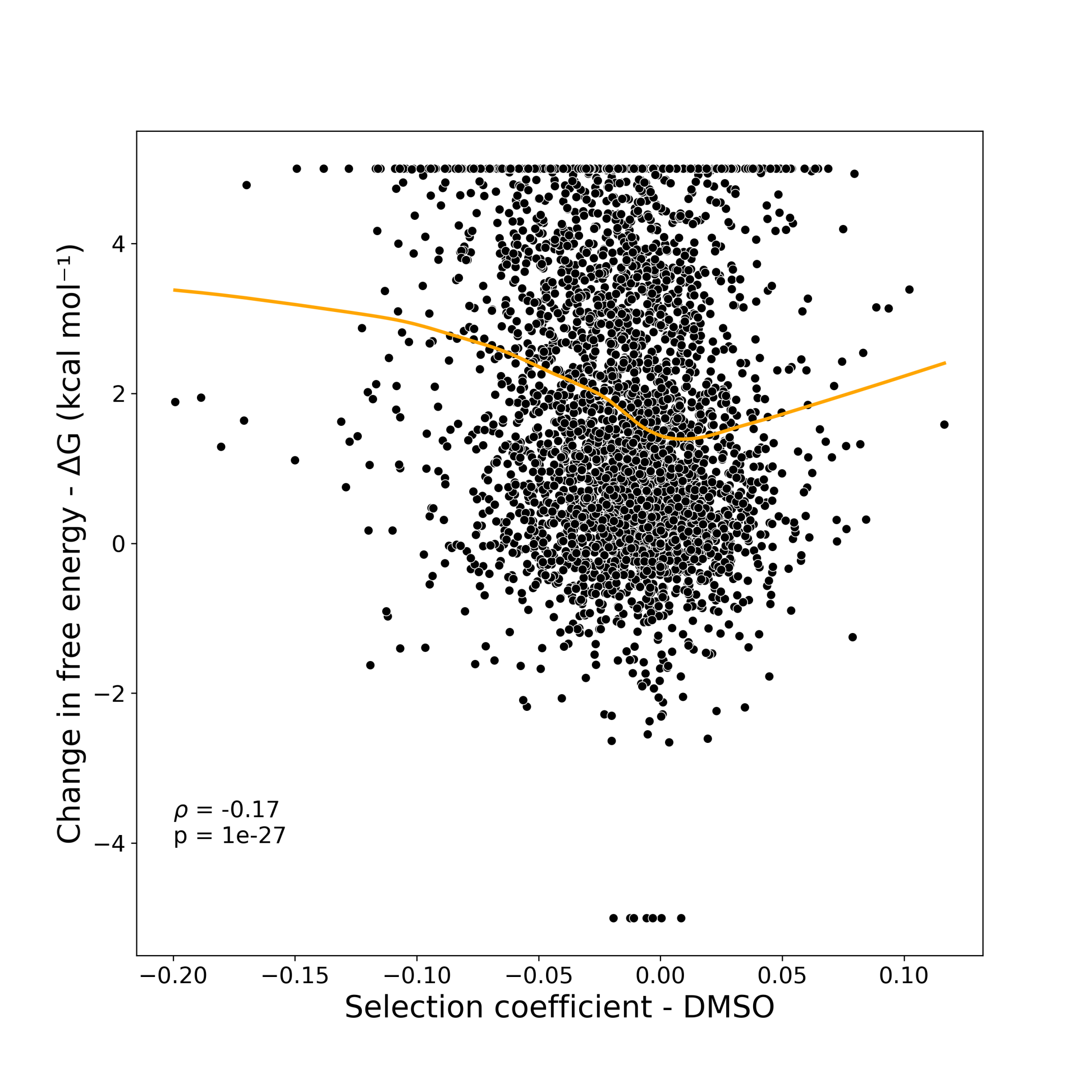

Supplement: S10 Fig — Using FoldX, changes in protein free energy were computed for all mutants forming a complex with DHF and compared to their selection coefficients in DMSO. Extreme values (-5<x<5 change in free energy) were scaled down to 5/-5, as extreme values of ddG measured by FoldX can often be attributed to clashes. Orange line represents the Lowess smoothed curve fitted using statsmodels default settings. Statistical tests are Spearman’s rank correlation. (TIFF) [file pgen.1011252.s010.tiff]

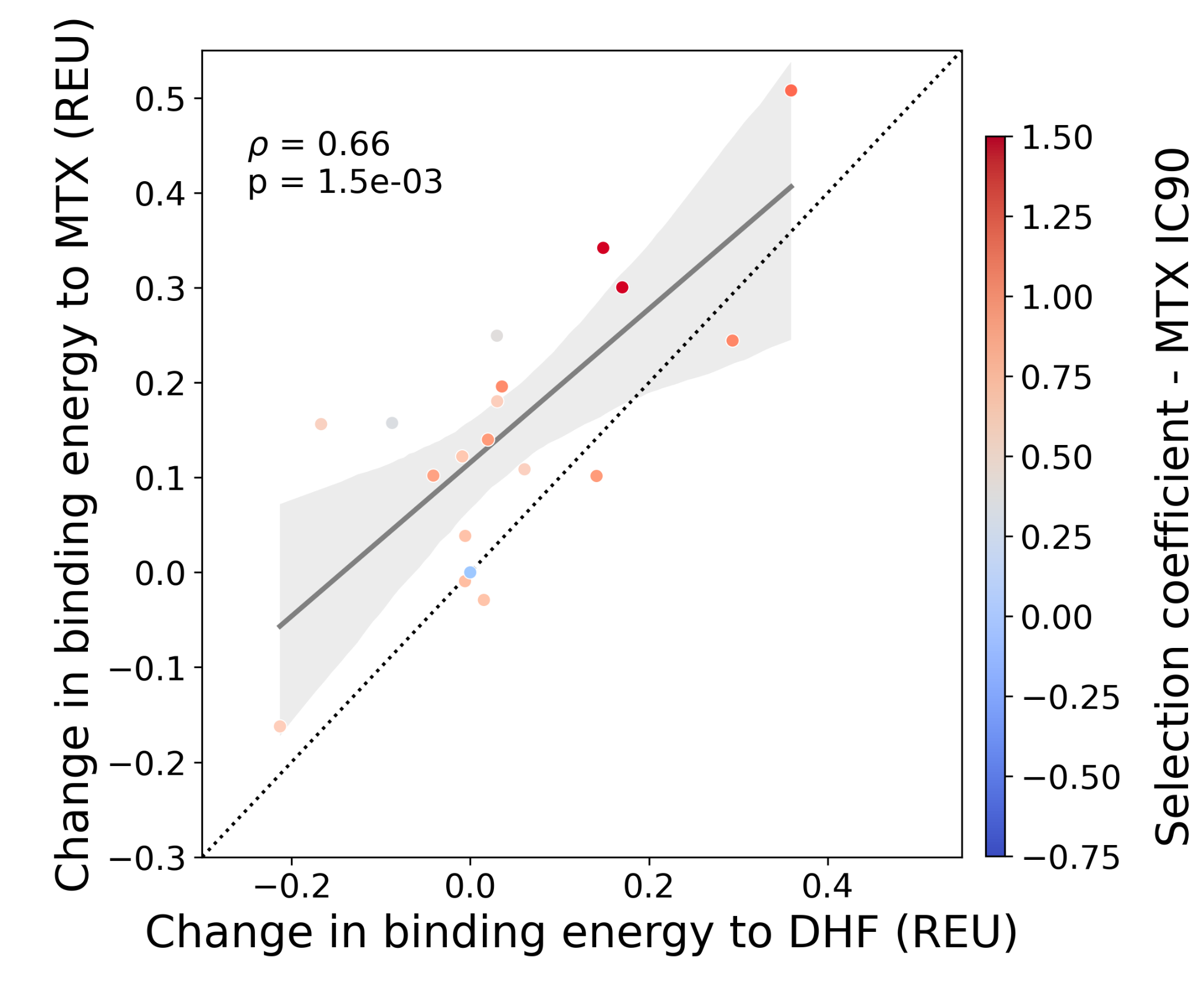

Supplement: S11 Fig — On the x-axis, we plot the change in binding energy between F199 mutants and DHF, and on the y-axis, between F199 mutants and MTX. The diagonal shows equal changes in binding energy to both molecules. Units are REU (Rosetta Energy Units). Statistical tests are Spearman’s rank correlation. (TIFF) [file pgen.1011252.s011.tiff]

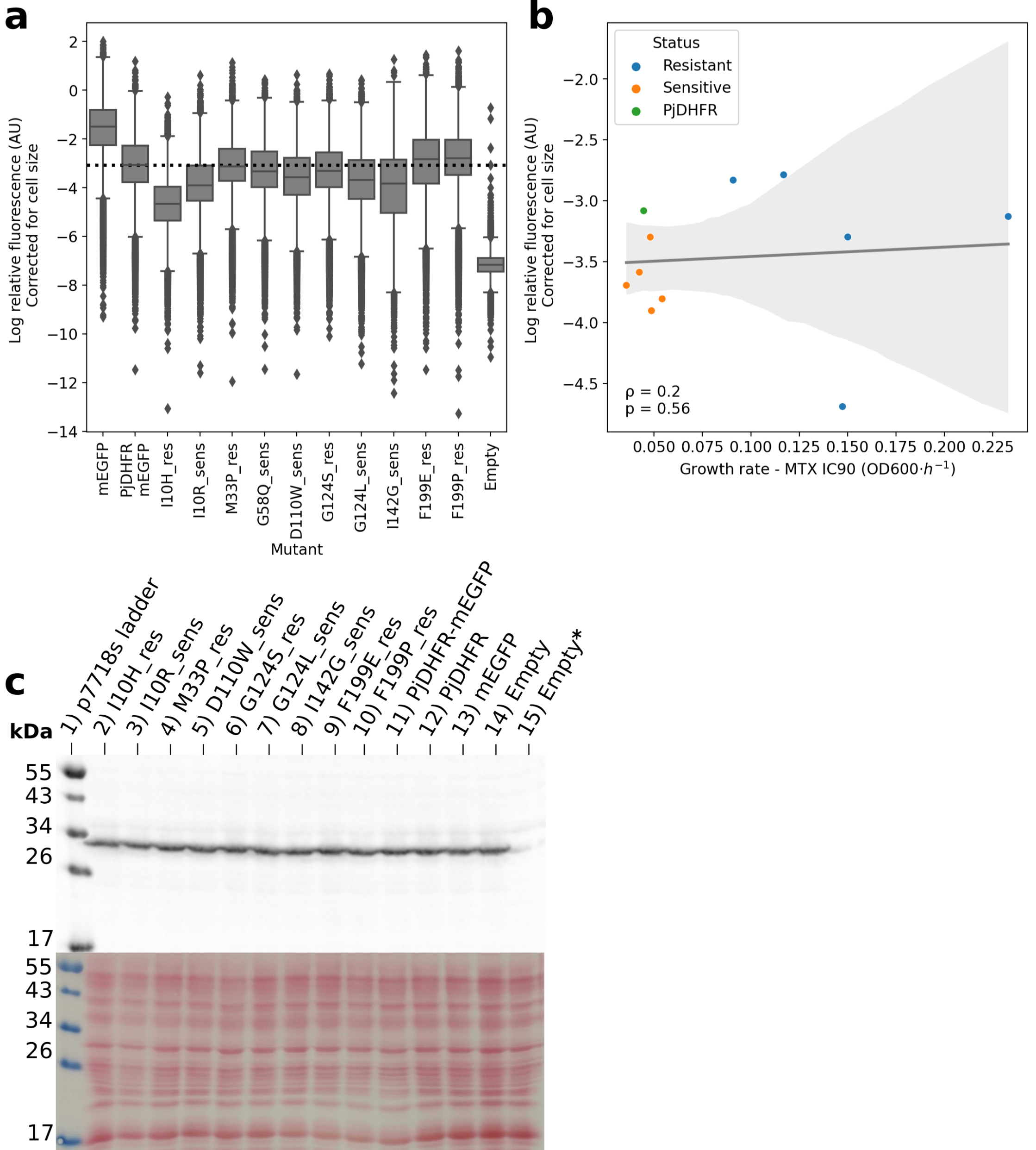

Supplement: S12 Fig — a) Flow cytometry measurements for different validation mutants and controls in strain IGA130. mEGFP represents mEGFP alone expressed under the regulation of the same promoter used for the screen. PjDHFR-mEGFP is the wild-type PjDHFR tagged with mEGFP. Mutants marked with sens or res are mutants that are identified as sensitive or resistant, respectively. Empty is the empty plasmid as a negative control. b) Correlation between median measured fluorescence and growth rate in MTX IC90, colored by mutant status. No correlation was found between expression levels and resistance. c) Anti-FLAG western blot and Ponceau red stains of strain FDR0003 (DFR1 3xFLAG-tag) to measure changes in expression levels of genomic DFR1 when co-expressed with PjDHFR mutants. 11) is PjDHFR-mEGFP fusion protein, 12) PjDHFR is PjDHFR wild-type without fusion, 13) is mEGFP without fusion, and 14) is Empty plasmid. All transformations were done in strain FDR0003 unless specified. 15) Empty* is empty plasmid transformed in strain IGA130 as a FLAG-negative control. (TIFF) [file pgen.1011252.s012.tiff]
