# Supplementary material for: Deep mutational scanning of Pneumocystis jirovecii dihydrofolate reductase reveals allosteric mechanism of resistance to an antifolate
Source: PLoS Genet. 2024 Apr 29;20(4):e1011252. doi: 10.1371/journal.pgen.1011252 (PMC11125491; doi:10.1371/journal.pgen.1011252)
Supplement: Attachment — Submitted filename: Papier_PjDHFR_PLOSGen_Response.pdf [file pgen.1011252.s023.pdf]

Dear Dr. Landry

Thank you very much for submitting your Research Article entitled 'Deep mutational scanning of *Pneumocystis jirovecii* dihydrofolate reductase reveals allosteric mechanism of resistance to an antifolate' to PLOS Genetics.

Your manuscript has been reviewed by 3 experts in microbial and protein evolution, and experts on DHFR. Overall, there is a consensus in the comments, both regarding impact and revisions suggested. Specifically, the following points need to be addressed:

0.1) Clearer explanation of the validity and caveats of the selection system for the deep mutational scan, particularly the use of MTX vs. TMP. See particularly the comments of Reviewer 3.

0.1 Response: *For our choice of using MTX instead of TMP for this screening, we had initially tried to measure inhibitory concentrations for TMP in this system. However, even at concentrations 10x higher than the inhibitory concentration that we use to inhibit growth of E. coli in the lab, neither strains used in this study (IGA130 and FDR0001) showed any inhibition by TMP (see ReviewFig1). We therefore had to select a different antifolate than TMP for this screening, as in accordance to literature, S. cerevisiae appeared to be insensitive to TMP through DHFR independent mechanisms.*

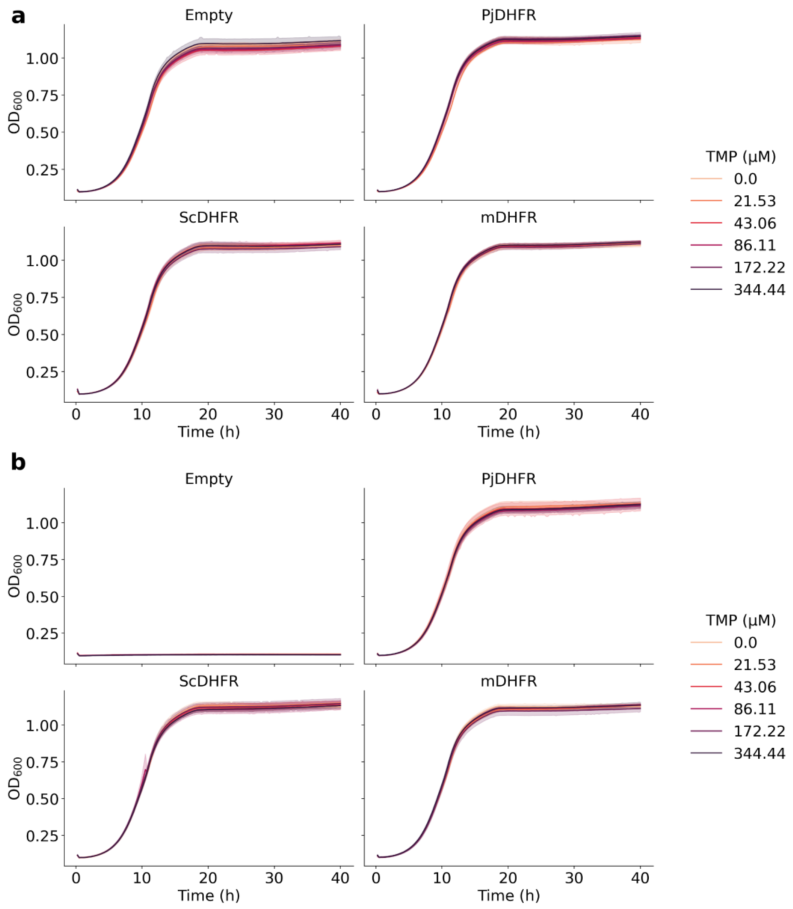

ReviewFig1: Dose response curve measurements for strain IGA130 and FDR0001 in the presence of TMP. a) *Growth curves of yeast strain IGA130 (wild-type for DFR1) each expressing a different DHFR. Shaded areas represent confidence intervals across biological triplicates. The strain appears to be insensitive to TMP regardless of the DHFR present. Measured IC50 for strain IGA130 with the Empty plasmid is ~17mg/mL, which is above the solubility limit of TMP in ethanol (~12mg/mL at RT) [1].* ***b)*** *Growth curves of yeast strain FDR0001 (dfr1Δ) each expressing a different DHFR. Shaded areas represent confidence intervals across biological triplicates. The strain appears to be insensitive to TMP regardless of the DHFR present.*

Based on our previous tests, as well as literature establishing a link between the use of MTX in clinical settings and the emergence of TMP resistance, we elected to use MTX for this screening. While it would have been optimal to be able to use TMP in this system, we believed that MTX was the best functional option available to us, as clinical links have been established in the evolution of TMP resistance based on MTX exposure [2], as well as the fact that we could get complete inhibition of PjDHFR using MTX even at high expression levels, highlighting the evolvability of this protein towards resistance.

Furthermore, to address the reason why we used strain IGA130 instead of FDR0001, we conducted dose response curves in strain FDR0001 in the same way we did for strain IGA130. The difference in IC50 (2.03µg/mL in IGA130 and 1.95µg/mL in FDR0001 for PjDHFR), was minimal, highlighting the small effect of genomic DFR1 on resistance to MTX (see ReviewFig2). Furthermore, while conducting validation growth curves in FDR0001, we observed that several mutations that were identified as being sensitive in the initial screen actually produced non-functional proteins. If the screen had been done in FDR0001, selection against non-functional DHFRs would have occurred at the library transformation step, which could have reduced the diversity of mutations available to us during downstream screening. In the light of these results, we believe that for our aim of screening all possible mutations in PjDHFR for MTX resistance, using strain IGA130 was the optimal choice available to us.

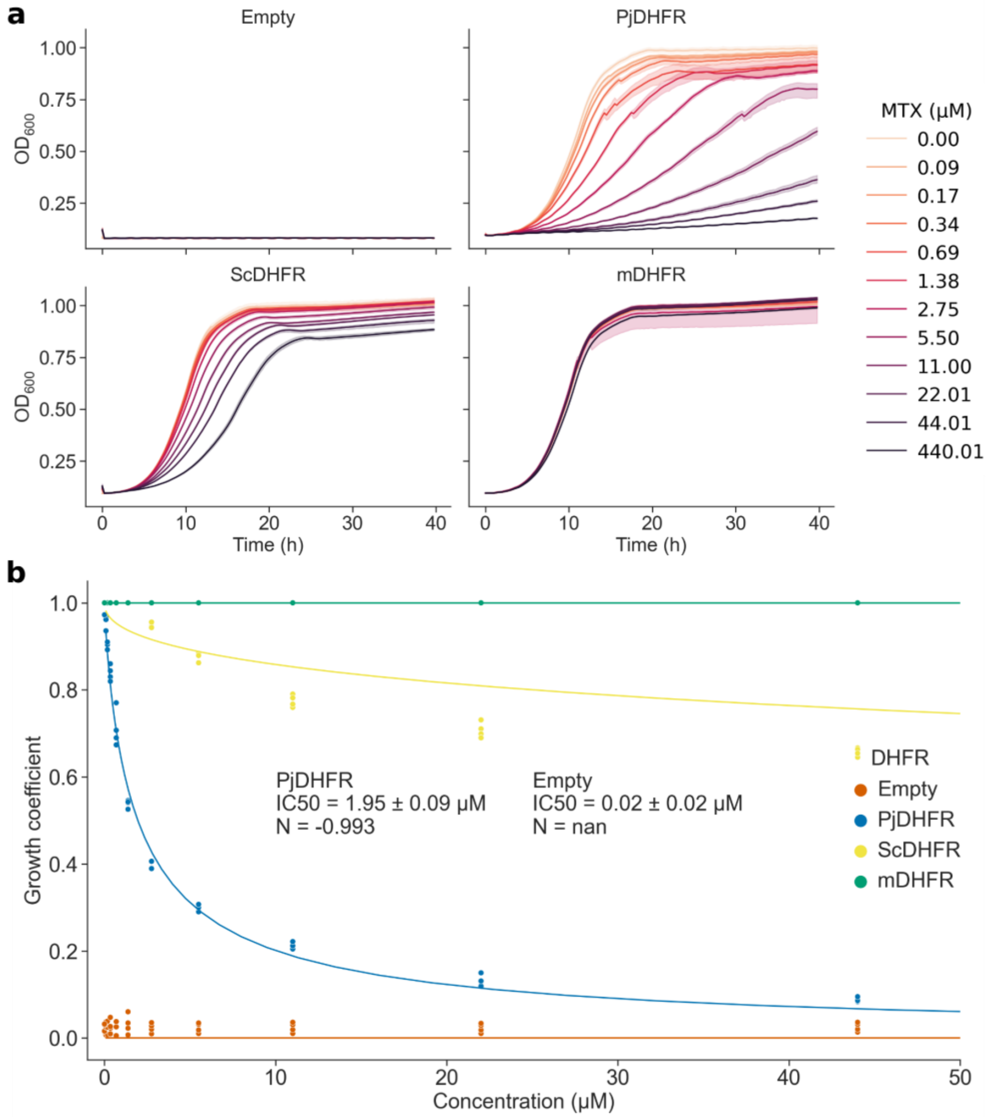

ReviewFig2: Dose response curve measurements for strain FDR0001 in the presence of MTX. **a)** Growth curves of yeast strain FDR0001 (*dfr1Δ*) each expressing a different DHFR. Shaded areas represent confidence intervals across biological triplicates. The Empty vector does not support growth in this strain. Strains with vector-expressed DHFRs show increased resistance, with PjDHFR being the most sensitive, followed by ScDHFR, and mDHFR(L22F/F31S) being fully resistant. **b)** Growth coefficient of strain FDR0001 with different DHFRs. R^2 for Hill equation fit: Empty = null, PjDHFR = 0.99, ScDHFR = 0.73 and mDHFR = >0.99.

This figure is now figure S3 in the manuscript.

Modifications in main text about caveats of the system (drugs and strains):

Lines 137-147 - Introduction: We chose MTX as our antifolate of choice for this screening as *S. cerevisiae* has been previously reported as being insensitive to TMP through mechanisms not directly involving the DHFR [45]. However, a recent study has associated the exposure of MTX in bacteria to both the selection for, and *de novo* evolution of, TMP resistance in bacteria [46]. Additionally, MTX is often used to treat auto-immune

diseases, and can act as an immunosuppressant [47], and has been associated with increased rates of *P. jirovecii* infection [48–53]. There is therefore a need to understand how MTX affects *P. jirovecii's* DHFR, and the link to resistance between these two antifolates. Finally, studying MTX resistance in the context of PjDHFR provides a tractable model for the study of underlying mechanisms of resistance-causing mutations in this protein.

Lines 555-561 - Results: To ensure that the use of strain IGA130 (wild-type for *DFR1*) did not introduce a bias in selection strength, we conducted identical dose response curves in strain FDR0001 (*dfr1Δ*) and estimated its IC50 as well (S3A, S3B Fig). IC50 measured for strain FDR0001 is of 1.95 μM, validating results from strain IGA130 on the minimal effect of genomic DFR1 on MTX resistance. We believe that by using this system, we will be able to identify a maximum number of mutations in PjDHFR that confer resistance to MTX, without introducing downstream bias for PjDHFR activity.

Lines 667-672 - Results: We then used these results to correlate growth rate in IGA130 and in FDR0001 to validate how the results from the high-throughput assay could be transposed to strain FDR0001 (*dfr1Δ*). In DMSO, all mutants in strain IGA130 grew similarity, but non-functional PjDHFR mutants in strain FDR0001 were not able to grow. In the presence of MTX, the phenotype of resistant mutants are very well correlated between the two strains, highlighting the robustness of this assay (S8C and S8D Fig).

Lines 887-890 - Discussion: While this system does not allow the direct investigation of TMP resistance, we believe that in the light of the clinical and evolutionary link between MTX, TMP and *P. jirovecii* infection, this study provides relevant insights in the evolution of antifolate resistance, and on the biophysics of MTX resistance in DHFRs.

Lines 899-903 - Discussion: While individual validations in strain FDR0001 (*dfr1Δ*) allowed to identify resistance/function tradeoffs, the high-throughput screening in strain IGA130 (wild-type for *DFR1*) did not allow systematic investigation of this relationship. Further experiments will be necessary to understand the functional tradeoff associated with MTX resistance in PjDHFR.

0.2) Discussion on the use of MTX for PjDHFR in the clinic, if there is, to broaden the impact of the work.

<u>0.2 Response</u>: *While MTX is not directly used to treat P. jirovecii infections, links have been established between the use of MTX and increased rates of P. jirovecii infection, as well as selection for TMP resistance in bacteria exposed to MTX in vitro [2–4]. We therefore believe that understanding the mechanisms of MTX resistance in PjDHFR will be helpful in predicting the outcomes of mutations on TMP resistance using computational models further down the line. The main text has been modified to expand on the link between MTX, TMP and P. jirovecii infection.*

<u>Modifications in main text about the relationship between MTX and P. jirovecii:</u>

Lines 137-147 - Introduction: We chose MTX as our antifolate of choice for this screening as *S. cerevisiae* has been previously reported as being insensitive to TMP through mechanisms not directly involving the DHFR [45]. However, a recent study has associated the exposure of MTX in bacteria to both the selection for, and *de novo* evolution of, TMP resistance in bacteria [46]. Additionally, MTX is often used to treat auto-immune diseases, and can act as an immunosuppressant [47], and has been associated with increased rates of *P. jirovecii* infection [48–53]. There is therefore a need to understand how MTX affects *P. jirovecci's* DHFR, and the link to resistance between these two antifolates. Finally, studying MTX resistance in the context of PjDHFR provides a tractable model for the study of underlying mechanisms of resistance-causing mutations in this protein.

Lines 933-938 - Discussion: However, as MTX is not used to treat *P. jirovecii* infections in clinical settings, and because of the conflicting reports of the effects of MTX on the evolution TMP resistance and on *P. jirovecii* infection, it is important to be critical about the nature of these results. We believe, however, that this is a step in the right direction, and that this data will help future studies focusing on more clinically relevant antifolates.

0.3) Two reviewers cited alternative explanations for the observed mutations (e.g., position F199) than the one provided by the authors. The authors should provide further support for their argument or acknowledge the alternative explanation.

0.3 Response: *Two mechanisms were suggested by reviewers. Firstly, some mutations could cause the increase of expression of genomic DFR1, which can then lead to resistance. To address this, we conducted two different new sets of experiments. First, we conducted all validations in strain FDR0001, and correlated degree of MTX resistance in strain FDR0001 and in IGA130 to demonstrate that genomic DFR1 has minimal effect on resistance. With a Spearman correlation of 0.79 in growth rates between all validation mutants in MTX for both strains (p-value = 2.62e-11, ReviewFig 3), we believe that this was not significant as a mechanism of resistance in this experiment, and that this validates our choice of conducting the full screen in strain IGA130.*

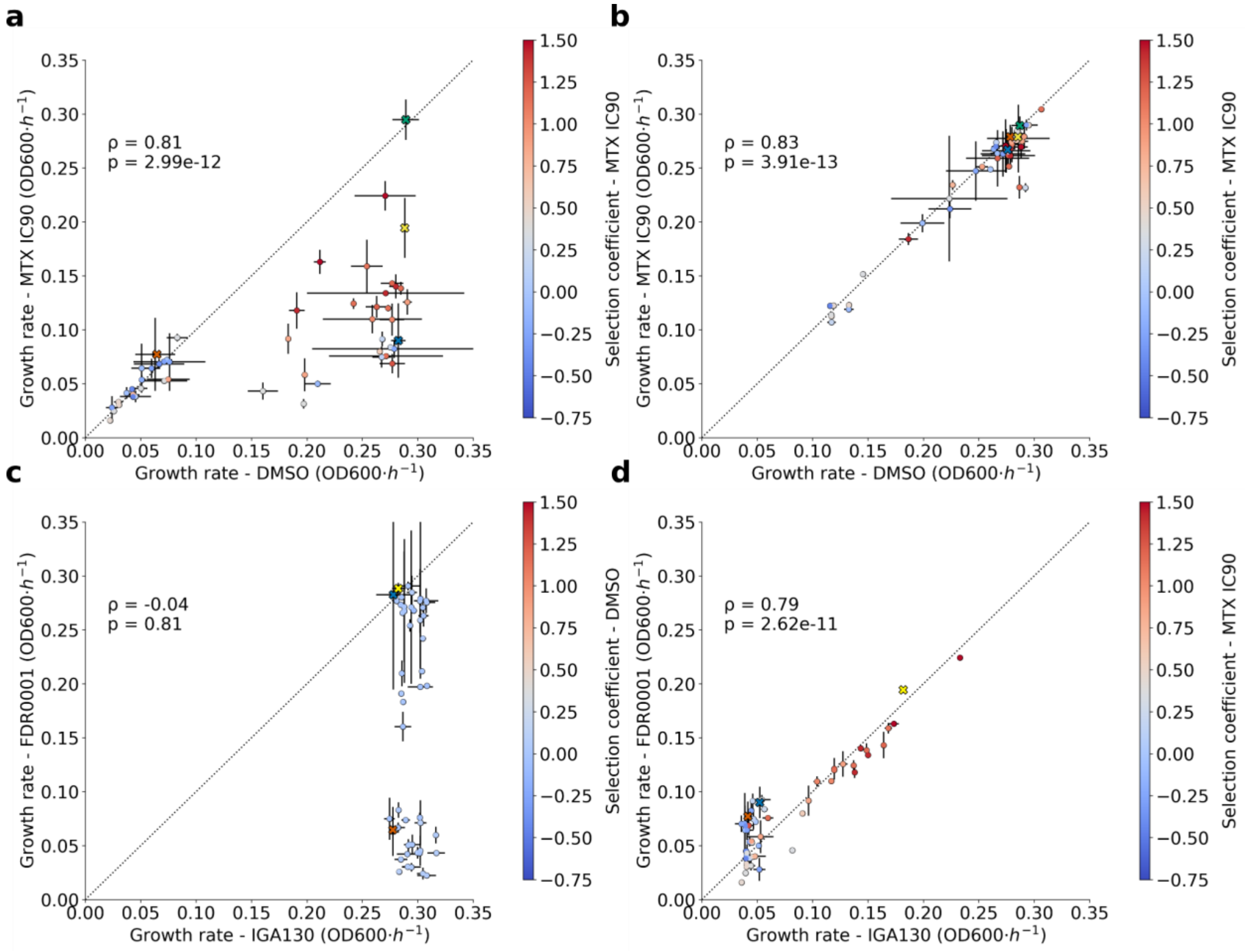

ReviewFig3**: Comparison of growth rates from validations growth curves in strain FDR0001 for some manually reconstructed mutants. a)** Growth rates in DMSO are plotted on the X axis, as a measure of complementation, and growth rates in the presence of MTX IC90 are plotted on the Y axis, as a measure of resistance. These growth curves were conducted in the absence of β-estradiol. Many sensitive mutants cannot complement the deletion of *DFR1* in strain FDR0001 (*dfr1Δ*). Error bars represent growth rate values measured across the biological replicates in both conditions. Data points marked by crosses represent the Empty plasmid (orange), the wild-type PjDHFR (blue), ScDHFR (yellow) and mDHFR (green). Other round data points are colored by their selection coefficients at IC90 unless specified otherwise. **b)** Same as panel a), but in the presence of β-estradiol, which leads to expression of MTX insensitive DfrB1. **c)** Comparison of growth rates between strains IGA130 (x-axis) and FDR0001 (y-axis) validation mutants in DMSO. All mutants can grow similarly in strain IGA130, but some mutants were non-functional in strain FDR0001. Color bar is the selection coefficient in DMSO. **d)** Comparison of growth rates between strains IGA130 (x-axis) and FDR0001 (y-axis) validation mutants in MTX IC90. For mutants that were identified as resistant, there is a strong correlation between growth rates measured in strain IGA130 and FDR0001, highlighting the minimal effect of the genomic DFR1 on MTX resistance. Non-functional mutants in FDR0001 had to be supplemented with 100nM β-estradiol to ensure growth of precultures. Statistical tests are Spearman's rank correlation.

This figure is now Figure S8 in the revised manuscript.

To further address this, we constructed strain FDR0003, where genomic DFR1 was tagged with protein purification 3xFLAG-tag, as well as plasmids with different mutants tagged with mEGFP. By conducting anti-FLAG and anti-mEGFP western blots, we were able to verify that DFR1 expression was not significantly increased between wild-type

mEGFP and the different mutants (See ReviewFig4C).

The second suggested mechanism was that some mutations increased protein stability, and therefore abundance, which could lead to increased resistance. This is a well-documented mechanism of resistance in DHFRs. To investigate this, we constructed some PjDHFR-mEGFP fusions using some of the validation mutants, and measured fluorescence intensity using flow cytometry as a proxy for protein abundance (5000 events, in triplicate). We did not observe a correlation between resistance and protein abundance (see ReviewFig4A and B). Mutants I10H and I10R (selection coefficients of 1.154 and 0.014, respectively) both had reduced abundance (0.71- and 0.82-fold change in relative fluorescence intensity per cell, respectively.) As for position 199 specifically, we had only reconstructed two validation mutants (F199P and F199E, selection coefficients of 1.018 and 0.585, respectively, both resistant since no mutations at position F199 leads to a negative selection coefficient.) These two mutants showed slightly increased expression levels, but the measured increase in expression is not proportional with the measured selection coefficient (F199P and F199E, with increase of 1.05- and 1.02-fold-change in relative fluorescence intensity per cell, respectively). Based on this evidence, we believe that MTX resistance cannot be solely attributed to increase in protein abundance, but it is possible that it is a contributing factor. The main text has been modified to reflect all these alternative mechanisms.

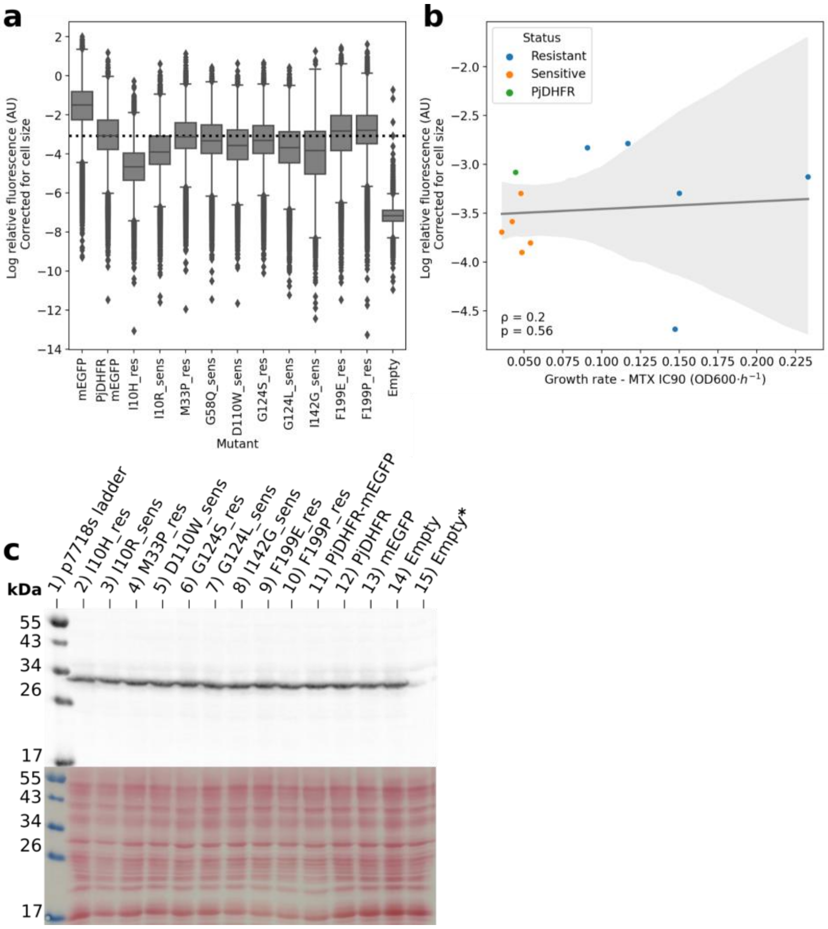

ReviewFig4: Measurements of fluorescence intensity of PjDHFR mutants tagged with mEGFP as a proxy for protein abundance. **a)** Flow cytometry measurements for different validation mutants and controls in strain IGA130. mEGFP represents mEGFP alone expressed under the regulation of the same promoter used for the screen. PjDHFR-mEGFP is the wild-type PjDHFR tagged with mEGFP. Mutants marked with sens or res are mutants that are identified as sensitive or resistant, respectively. Empty is the empty plasmid as a negative control. **b)** Correlation between median measured fluorescence and growth rate in MTX IC90, collared by mutant status. No correlation was found between expression levels and resistance. **c)** Anti-FLAG western blot and Ponceau S red stains of strain FDR0003 (DFR1 3xFLAG-tag) to measure changes in expression levels of genomic DFR1 when co-expressed with PjDHFR mutants. 11) is PjDHFR-mEGFP fusion protein, 12) PjDHFR is PjDHFR wild-type without fusion, 13) is mEGFP without fusion, and 14) is Empty plasmid. All transformations were done in strain FDR0003 unless specified. 15) Empty***** is Empty plasmid transformed in strain IGA130 as a FLAG-negative control.

This figure is now figure S12 in the revised manuscript.

<u>Modifications in main text about alternative mechanisms of resistance:</u>

Lines 820-822 - Results: Position F199 is also in close proximity to the side chain of position F35, and could be forming π-stacking interactions with this other phenylalanine, which could then affect the α-helix where position M33 is located [81].

Lines 850-877 - Results: **Alternative mechanisms of MTX resistance in PjDHFR**

Alternative mechanisms of resistance were also investigated. Indeed, antifolate resistance caused by increased DHFR expression or protein abundance has been documented. To verify this, we constructed PjDHFR-mEGFP fusion proteins for wild-type PjDHFR and some validation mutants of interest. Fluorescence intensity was measured by flow cytometry as a proxy for protein abundance (S12A Fig.). As all mutants are under the regulation of the same promoter, variation in abundance should depend on changes in protein stability caused by the mutations. We did not observe a correlation between resistance and protein abundance (S12B Fig.). Mutants I10H and I10R (selection coefficients of 1.154 and 0.014, respectively) both had reduced abundance (0.71 and 0.82 fold change in relative fluorescence intensity per cell, respectively), where the most resistant mutant of the two is the least abundant. As for position 199 specifically, we had only reconstructed two validation mutants (F199P and F199E, selection coefficients of 1.018 and 0.585, respectively, both resistant, since no mutations at position F199 leads to a negative selection coefficient). These two mutants showed slightly increased expression levels, but the measured increase in expression is not proportional with the measured selection coefficient (F199P and F199E, with increase of 1.05 and 1.02 fold-change in relative fluorescence intensity per cell, respectively). Based on these observations, we believe that MTX resistance cannot be solely attributed to increase in protein abundance, but it is possible that it is a contributing factor.

As the screen was conducted in strain IGA130 (wild-type for genomic DFR1), it was also possible that the expression of PjDHFR mutants from the plasmid caused fluctuations in the expression of genomic DFR1, affecting measured DMS scores. To test this, we constructed strain FDR0003, which is the same as strain IGA130, but where genomic DFR1 is tagged with 3xFLAG-tag in C-terminal. We measured changes in genomic DFR1 expression by anti-FLAG western blot in the presence of several resistant and sensitive

mutants, and did not observe any changes in DFR1 expression in the presence of PjDHFR mutants (S12C Fig.)

Lines 927-931 - Discussion: While protein abundance measurements showed no clear correlation with resistance in this study, increase in protein stability has been well documented as an antifolate resistance mechanism, and at least two of the tested resistant mutants showed increase in protein abundance.

Should you decide to revise the manuscript for further consideration here, your revisions should address the specific points made by each reviewer. We will also require a detailed list of your responses to the review comments and a description of the changes you have made in the manuscript.

If you decide to revise the manuscript for further consideration at PLOS Genetics, please aim to resubmit within the next 60 days, unless it will take extra time to address the concerns of the reviewers, in which case we would appreciate an expected resubmission date by email to plosgenetics@plos.org.

If present, accompanying reviewer attachments are included with this email; please notify the journal office if any appear to be missing. They will also be available for download from the link below. You can use this link to log into the system when you are ready to submit a revised version, having first consulted our Submission Checklist.

To enhance the reproducibility of your results, we recommend that you deposit your laboratory protocols in protocols.io, where a protocol can be assigned its own identifier (DOI) such that it can be cited independently in the future. Additionally, PLOS ONE offers an option to publish peer-reviewed clinical study protocols. Read more information on sharing protocols at https://plos.org/protocols?utm_medium=editorial-email&utm_source=authorletters&utm_campaign=protocols

Please be aware that our data availability policy requires that all numerical data underlying graphs or summary statistics are included with the submission, and you will need to provide this upon resubmission if not already present. In addition, we do not permit the inclusion of phrases such as "data not shown" or "unpublished results" in manuscripts. All points should be backed up by data provided with the submission.

While revising your submission, please upload your figure files to the Preflight Analysis and Conversion Engine (PACE) digital diagnostic tool. PACE helps ensure that figures meet PLOS requirements. To use PACE, you must first register as a user. Then, login and navigate to the UPLOAD tab, where you will find detailed instructions on how to use the tool. If you encounter any issues or have any questions when using PACE, please email us at figures@plos.org.

PLOS has incorporated Similarity Check, powered by iThenticate, into its journal-wide submission system in order to screen submitted content for originality before publication. Each PLOS journal undertakes screening on a proportion of submitted articles. You will be contacted if needed following the screening process.

To resubmit, use the link below and 'Revise Submission' in the 'Submissions Needing

Revision' folder.

We are sorry that we cannot be more positive about your manuscript at this stage. Please do not hesitate to contact us if you have any concerns or questions.

Yours sincerely,

Adrian Serohijos, PhD
Guest Editor
PLOS Genetics

Geraldine Butler
Section Editor
PLOS Genetics

Reviewer's Responses to Questions

Comments to the Authors:

Please note here if the review is uploaded as an attachment.

Reviewer #1: Rouleau et al present a thorough study of methotrexate resistance mutations in Pneumocystis jirovecii DHFR, a potential drug target in a hard-to-study fungal pathogen. They use deep mutational scanning to comprehensively screen for resistance-causing mutations in two drug concentrations, and identify 355 mutations at 31 positions associated with resistance. One interesting finding is several seemingly “allosteric” mutations which result in resistance though they are located away from the active site. The paper is well written and exceedingly clear. It presents new tools for studying resistance in an important pathogen, and will be of strong relevance to geneticists and protein biochemists with interests in drug resistance. I was impressed with the rigor of the statistical analysis and the organization of the associated github repository, which together make the data processing transparent. In some respects the functional assay design seems suboptimal (which somewhat diminishes by enthusiasm for the work, see comments below), but the results presented are on the whole intriguing and well supported by the data.

Major comments:

1.1. It was not clear to me from the introduction if: (A) methotrexate is a common clinical treatment of P. jirovecii, with resistance an emerging threat, (B) trimethoprim (which would surely be less toxic) is the more commonly used clinical treatment, but didn’t work in their screen, hence they pivoted to MTX as another antifolate, or (C) antifolates as a whole aren’t usually used in P jirovecii treatment at present, but are under consideration so it is important to know how easy it is to evolve resistance. Which is the case? More explicit clarification on the connection between this work and current clinical practice (and potential future applications/impact of this assay – what would/could you screen next? Would/could you use this assay in drug design?) would help in gauging the impact of this work.

1.1 Response: *Indeed, TMP-SFX is the most commonly used clinical treatment. However, our initial tests with TMP showed that S. cerevisiae appears to be insensitive to TMP in a DHFR independent manner (See ResponseFig1). Neither strain IGA130 nor FDR0001 with any of the DHFR showed sensitivity to TMP, even at concentrations 10x greater than what is used to inhibit E. coli growth. However, links have been established between the use of MTX and increased rates of P. jirovecii infection, as well as selection for TMP resistance in bacteria exposed to MTX in vitro [2–4]. We therefore believe that the study of the evolution of resistance to MTX in PjDHFR could give insights on mechanisms of resistance to TMP, for which the direct study is currently not possible systematically using available systems.*

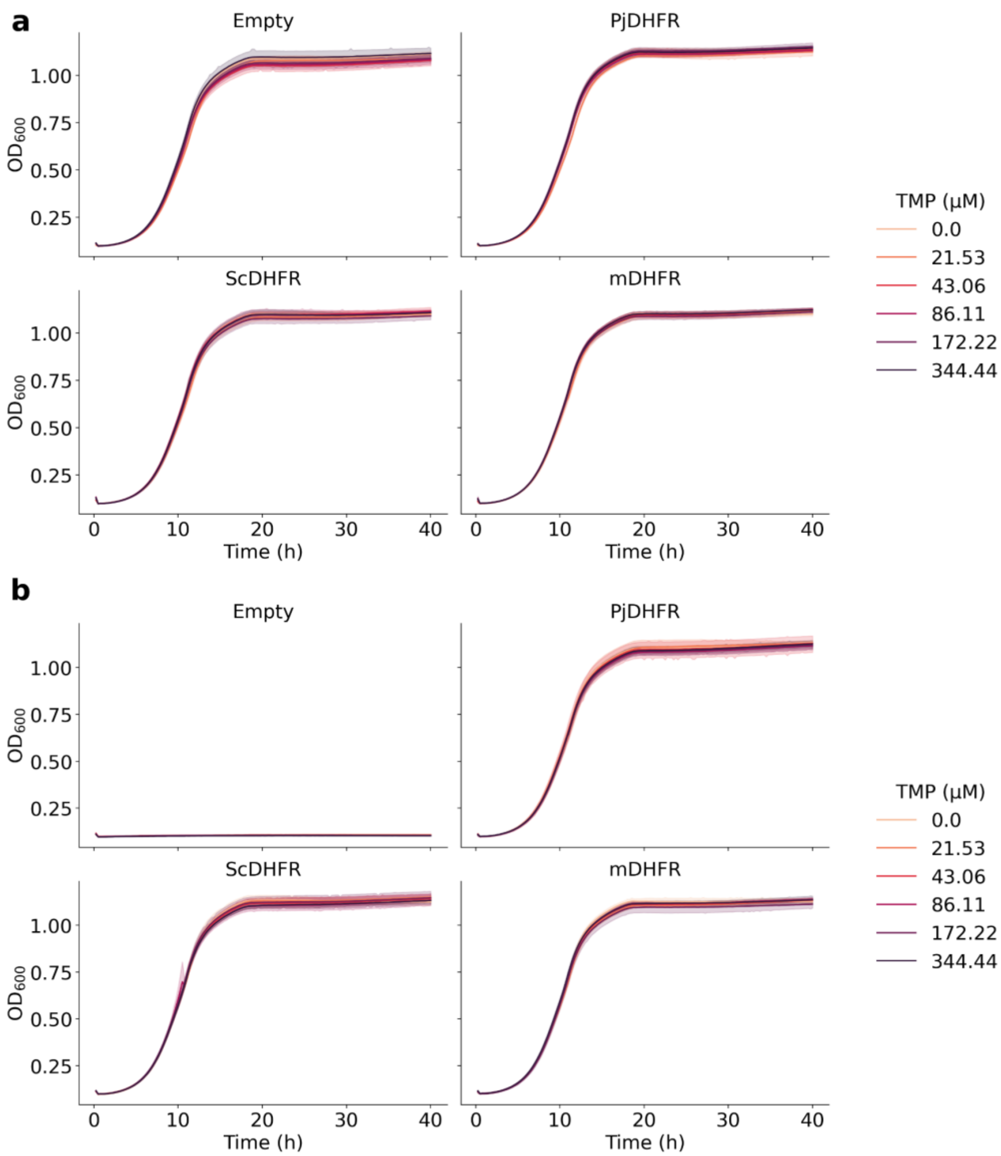

ReviewFig1: Dose response curve measurements for strain IGA130 and FDR0001 in the presence of TMP. a) *Growth curves of yeast strain IGA130 (wild-type for DFR1) each expressing a different DHFR. Shaded areas represent confidence intervals across biological triplicates. The strain appears to be insensitive to TMP regardless of the DHFR present. Measured IC50 for strain IGA130 with the Empty plasmid is ~17mg/mL, which is above the solubility limit of TMP in ethanol (~12mg/mL at RT) [1].* ***b)*** *Growth curves of yeast strain FDR0001 (dfr1Δ) each expressing a different DHFR. Shaded areas represent confidence intervals across biological triplicates. The strain appears to be insensitive to TMP regardless of the DHFR present.*

1.2. A central contribution of this paper is “an experimental system and a resource for the study of resistance mutations to antifolates in the DHFR of P. jirovecii (PjDHFR)”. However, there are several places where I wondered about the efficacy of the assay as a general tool. In particular:

(A) The authors mention that S. cerevisiae is insensitive to TMP through mechanisms not involving DHFR. Could this be a problem for other antifolates or classes of antifolates?

1.2.A Response: *Historically, antifolates have not been used to treat fungal infections clinically, except in the case of P. jirovecii infection. However, a recent study suggested the potential of using antifolates as antifungals, but highlighted the limited literature on the topic [5]. It is therefore difficult to infer if S. cereivisae's unknown mechanism of insensitivity to TMP can be generalized to other antifolates. However, other well characterized antifolates used in eukaryotic species, such as methotrexate, pyrimethamine and aminopterin, have been used in previous studies to inhibit S. cerevisiae DHFR, hinting that S. cerevisiae' insensitivity to TMP is a general mechanism of antifolate resistance [6,7].*

(B) The screen was done in the background of the endogenous S. cerevisiae DFR1 because the deletion strain had poor transformation efficiency. As a consequence, the current assay does not present a way to assess the impact of mutations on function in the absence of drug. For example, one can't tell from this assay if mutations at F31 show an activity-resistance tradeoff like the analogous mutations in other orthologs. Is there some way to address this in future work, or gauge this from existing data?

1.2.B Response*: *For validation mutants explicitly tested in strain FDR0001, mutations that affect protein function can be identified (see ReviewFig6). However, it is not possible to glean more information from the currently available high-throughput data, outside of systematic mutant reconstruction. We intend to explore this in a systematic manner in future work, where we will delve specifically into this resistance/function tradeoff. In the current study, our focus was on MTX resistance-causing mutations, and we did not systematically explore the biochemical impact of mutations beyond this, which is part of the reason why we conducted the screen in strain IGA130 (wild-type DFR1) and not in FDR0001 (dfr1Δ). In future works, we plan on conducting this screening in strain FDR0001 in the absence of β-estradiol, where protein function will be directly selected for, but this demands further optimization.*

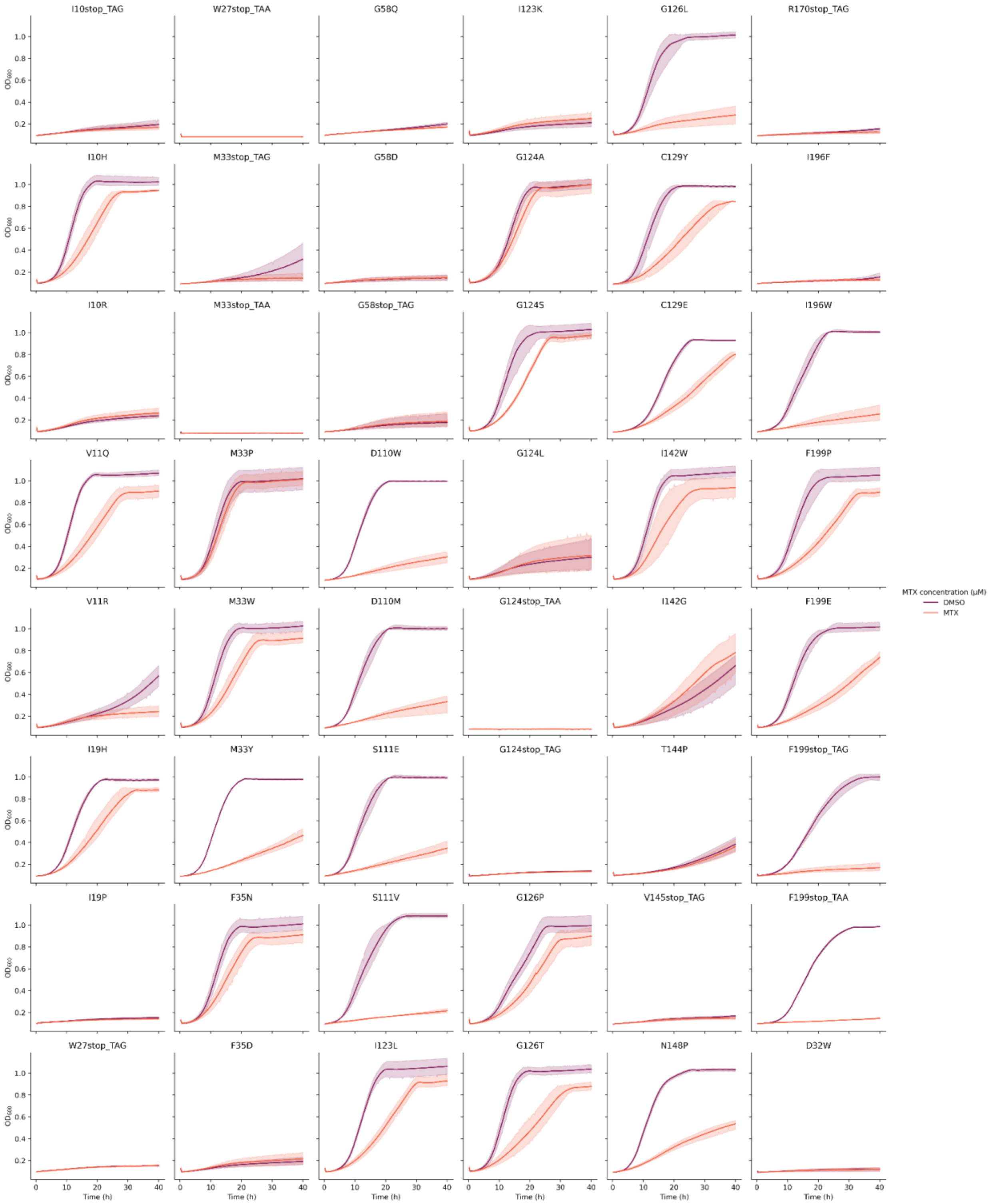

ReviewFig5: individual growth curves for validation of mutations in strain FDR0001 in DMSO and IC90. *Mutants are identified on top of their individual growth curves. For stop codons, we constructed both TAG (from DMS using NNK degenerate primers) and TAA as extra controls to ensure that TAG stop codons did not allow read through. All curves were done in triplicate, with error intervals between replicates appearing around the curve in a lighter color. Strains were grown in the same media as the competition assay for 40 hours, and validation growth rates were measured from these curves.*

Small discrepancies intervals in some of the curves in DMSO can be attributed to the formation of bubbles within the wells of the plates. These intervals were not considered when measuring growth rate.

Overall it would help if the authors could include some discussion of assay limitations and potential ways to overcome them in future work.

1.2 overall response: *The main text has been modified to include the different limitations inherent to this study. The main points addressed are the use of MTX instead of TMP, as well as the screen being conducted in a strain with a functional endogenous DHFR, and further comparisons have been made between validations in both strains. Thank you for your comments.*

Modifications in main text about caveats of the system (drugs and strains):

Lines 137-147 - Introduction: We chose MTX as our antifolate of choice for this screening as *S. cerevisiae* has been previously reported as being insensitive to TMP through mechanisms not directly involving the DHFR [45]. However, a recent study has associated the exposure of MTX in bacteria to both the selection for, and *de novo* evolution of, TMP resistance in bacteria [46]. Additionally, MTX is often used to treat auto-immune diseases, and can act as an immunosuppressant [47], and has been associated with increased rates of *P. jirovecii* infection [48–53]. There is therefore a need to understand how MTX affects *P. jirovecii's* DHFR, and the link to resistance between these two antifolates. Finally, studying MTX resistance in the context of PjDHFR provides a tractable model for the study of underlying mechanisms of resistance-causing mutations in this protein.

Lines 555-561 - Results: To ensure that the use of strain IGA130 (wild-type for *DFR1*) did not introduce a bias in selection strength, we conducted identical dose response curves in strain FDR0001 (*dfr1Δ*) and estimated its IC50 as well (S3A, S3B Fig). IC50 measured for strain FDR0001 is of 1.95 μM, validating results from strain IGA130 on the minimal effect of genomic DFR1 on MTX resistance. We believe that by using this system, we will be able to identify a maximum number of mutations in PjDHFR that confer resistance to MTX, without introducing downstream bias for PjDHFR activity.

Lines 667-672 - Results: We then used these results to correlate growth rate in IGA130 and in FDR0001 to validate how the results from the high-throughput assay could be transposed to strain FDR0001 (*dfr1Δ*). In DMSO, all mutants in strain IGA130 grew similarity, but non-functional PjDHFR mutants in strain FDR0001 were not able to grow. In the presence of MTX, the phenotype of resistant mutants are very well correlated between the two strains, highlighting the robustness of this assay (S8C and S8D Fig).

Lines 887-890 - Discussion: While this system does not allow the direct investigation of TMP resistance, we believe that in the light of the clinical and evolutionary link between MTX, TMP and *P. jirovecii* infection, this study provides relevant insights in the evolution of antifolate resistance, and on the biophysics of MTX resistance in DHFRs.

Lines 899-903 - Discussion: While individual validations in strain FDR0001 (*dfr1Δ*)

allowed to identify resistance/function tradeoffs, the high-throughput screening in strain IGA130 (wild-type for *DFR1*) did not allow systematic investigation of this relationship. Further experiments will be necessary to understand the functional tradeoff associated with MTX resistance in PjDHFR.

1.3. I really like the idea that mutations at I10, I42, and F199 are acting allosterically to impact MTX binding. But could it also be possible that these mutations are instead enhancing stability and increasing pjDHFR abundance overall inside the cell?

1.3 Response: *Indeed, some mutations could cause increased protein stability, and therefore abundance, which could lead to increased resistance. This is a well-documented mechanism of resistance in DHFRs [8]. To investigate this possibility, we constructed PjDHFR-mEGFP fusions using some of the validation mutants, and measured fluorescence intensity using flow cytometry as a proxy for protein abundance (5000 events, in triplicate). We did not observe a correlation between resistance and protein abundance (see ReviewFig5A and B). Mutants I10H and I10R (selection coefficients of 1.154 and 0.014, respectively) both had reduced abundance (0.71 and 0.82 fold change in relative fluorescence intensity per cell, respectively.) As for position 199 specifically, we had only reconstructed two validation mutants (F199P and F199E, selection coefficients of 1.018 and 0.585, respectively, both resistant since no mutations at position F199 leads to a negative selection coefficient.) These two mutants showed slightly increased expression levels, but the measured increase in expression is not proportional with the measured selection coefficient (F199P and F199E, with increase of 1.05 and 1.02 fold-change in relative fluorescence intensity per cell, respectively). Based on these observations, we believe that MTX resistance cannot be solely attributed to increase in protein abundance, but it is possible that it is a contributing factor. The main text has been modified to reflect this.*

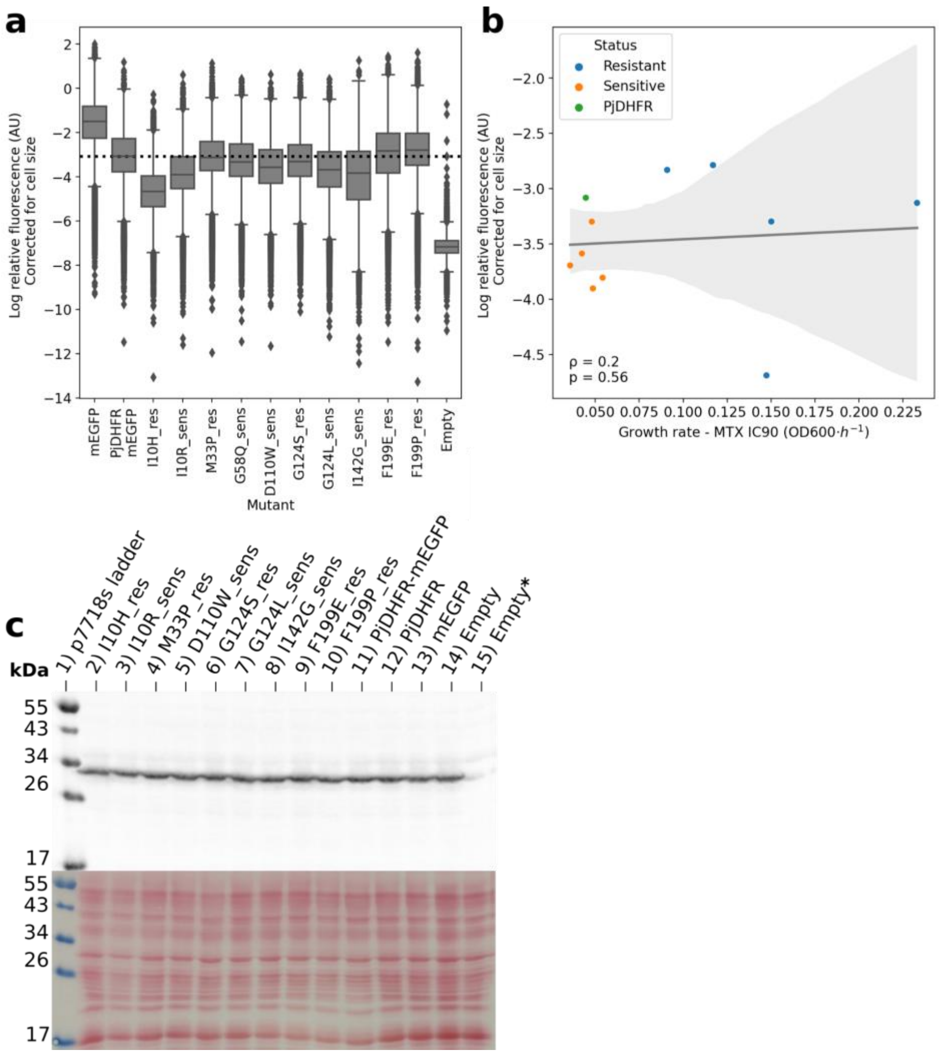

ReviewFig4: Measurements of fluorescence intensity of PjDHFR mutants tagged with mEGFP as a proxy for protein abundance. **a)** Flow cytometry measurements for different validation mutants and controls in strain IGA130. mEGFP represents mEGFP alone expressed under the regulation of the same promoter used for the screen. PjDHFR-mEGFP is the wild-type PjDHFR tagged with mEGFP. Mutants marked with sens or res are mutants that are identified as sensitive or resistant, respectively. Empty is the empty plasmid as a negative control. **b)** Correlation between median measured fluorescence and growth rate in MTX IC90, collared by mutant status. No correlation was found between expression levels and resistance. **c)** Anti-FLAG western blot and Ponceau S red stains of strain FDR0003 (DFR1 3xFLAG-tag) to measure changes in expression levels of genomic DFR1 when co-expressed with PjDHFR mutants. 11) is PjDHFR-mEGFP fusion protein, 12) PjDHFR is PjDHFR wild-type without fusion, 13) is mEGFP without fusion, and 14) is Empty plasmid. All transformations were done in strain FDR0003 unless specified. 15) Empty***** is Empty plasmid transformed in strain IGA130 as a FLAG-negative control.

This figure is now figure S12 in the revised manuscript.

<u>Modifications in main text about alternative mechanisms of resistance:</u>

Lines 820-822 - Results: Position F199 is also in close proximity to the side chain of position F35, and could be forming π-stacking interactions with this other phenylalanine, which could then affect the α-helix where position M33 is located [81].

Lines 850-877 - Results: **Alternative mechanisms of MTX resistance in PjDHFR**

Alternative mechanisms of resistance were also investigated. Indeed, antifolate resistance caused by increased DHFR expression or protein abundance has been documented. To verify this, we constructed PjDHFR-mEGFP fusion proteins for wild-type PjDHFR and some validation mutants of interest. Fluorescence intensity was measured by flow cytometry as a proxy for protein abundance (S12A Fig.). As all mutants are under the regulation of the same promoter, variation in abundance should depend on changes in protein stability caused by the mutations. We did not observe a correlation between resistance and protein abundance (S12B Fig.). Mutants I10H and I10R (selection coefficients of 1.154 and 0.014, respectively) both had reduced abundance (0.71 and 0.82 fold change in relative fluorescence intensity per cell, respectively), where the most resistant mutant of the two is the least abundant. As for position 199 specifically, we had only reconstructed two validation mutants (F199P and F199E, selection coefficients of 1.018 and 0.585, respectively, both resistant, since no mutations at position F199 leads to a negative selection coefficient). These two mutants showed slightly increased expression levels, but the measured increase in expression is not proportional with the measured selection coefficient (F199P and F199E, with increase of 1.05 and 1.02 fold-change in relative fluorescence intensity per cell, respectively). Based on these observations, we believe that MTX resistance cannot be solely attributed to increase in protein abundance, but it is possible that it is a contributing factor.

As the screen was conducted in strain IGA130 (wild-type for genomic DFR1), it was also possible that the expression of PjDHFR mutants from the plasmid caused fluctuations in the expression of genomic DFR1, affecting measured DMS scores. To test this, we constructed strain FDR0003, which is the same as strain IGA130, but where genomic DFR1 is tagged with 3xFLAG-tag in C-terminal. We measured changes in genomic DFR1 expression by anti-FLAG western blot in the presence of several resistant and sensitive mutants, and did not observe any changes in DFR1 expression in the presence of PjDHFR mutants (S12C Fig.)

Lines 927-931 - Discussion: While protein abundance measurements showed no clear correlation with resistance in this study, increase in protein stability has been well documented as an antifolate resistance mechanism, and at least two of the tested resistant mutants showed increase in protein abundance.

Minor comments:

Mi1.1. There seems to be a typo in equation 2, I believe the denominator in the second log () term should be median freq silent T_0 NOT T_final

<u>Minor1.1 Response</u>: *This was indeed a typographic mistake on our part. This has been fixed in the revised version. Thank you very much for the level of attention with which you read our manuscript.*

Mi1.2. Fig S2b, legend – line 1139, I think they mean alternative START codon, not STOP codon?

Minor1.2 Response: *We indeed meant an alternative START codon. This typo has been fixed in the revised version.*

2.1 Reviewer #2: In the manuscript by Rouleau et al. the authors perform saturating mutational studies on P. jirovecii's DHFR in yeast with the antifolate inhibitor and potent chemotherapeutic methotrexate (MTX). They chose methotrexate over trimethoprim (TMP) (an anti-infective DHFR inhibitor) as "S. cerevisiae has been reported as being insensitive to TMP through mechanisms not directly involving the DHFR". However TMP is ~40nM inhibitor where MTX appears to be micromolar. While this maybe what was experimentally tractable in their study and appears to be a carefully performed experiments, the impact is lessened significantly as therapeutic relevance against MTX is not justified or explained.

2.1 Response: *Indeed, TMP-MFX is the most commonly used clinical treatment. However, our initial tests with TMP showed that S. cerevisiae appears to be insensitive to TMP in a DHFR independent manner (See ResponseFig1). Neither strain IGA130 nor FDR0001 with any of the DHFR showed sensitivity to TMP, even at concentrations 10x greater than what is used to inhibit E. coli growth. However, links have been established between the use of MTX and increased rates of P. jirovecii infection, as well as selection for TMP resistance in bacteria exposed to MTX in vitro [2–4]. We therefore believe that the study of the evolution of resistance to MTX in PjDHFR could give insights on mechanisms of resistance to TMP, for which the direct study is currently not possible systematically using available systems.*

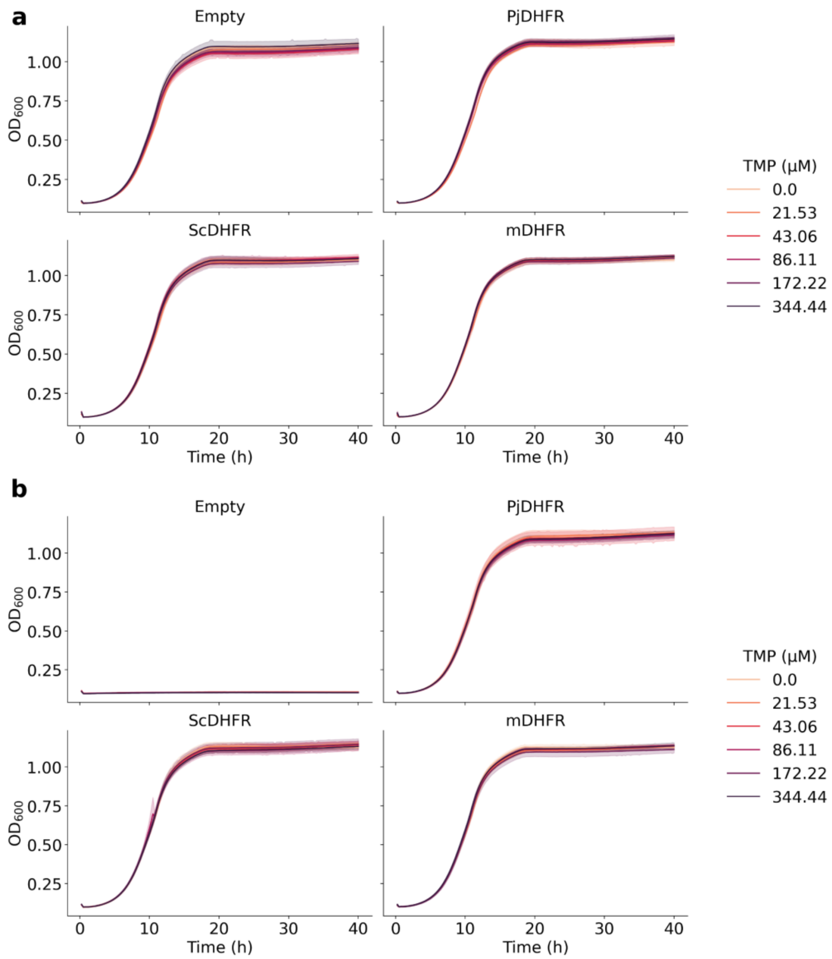

ReviewFig1: Dose response curve measurements for strain IGA130 and FDR0001 in the presence of TMP. a) *Growth curves of yeast strain IGA130 (wild-type for DFR1) each expressing*

a different DHFR. Shaded areas represent confidence intervals across biological triplicates. The strain appears to be insensitive to TMP regardless of the DHFR present. Measured IC50 for strain IGA130 with the Empty plasmid is ~17mg/mL, which is above the solubility limit of TMP in ethanol (~12mg/mL at RT) [1]. ***b)*** *Growth curves of yeast strain FDR0001 (dfr1Δ) each expressing a different DHFR. Shaded areas represent confidence intervals across biological triplicates. The strain appears to be insensitive to TMP regardless of the DHFR present.*

2.2 In fact, a recent study Antimicrob Agents Chemother. 2022 Dec; 66(12): e00990-22. doi: 10.1128/aac.00990-22, PMCID: PMC9765006, PMID: 36317930 has shown that patients undergoing therapy with MTX do not get relief from P. jirovecii infections and resistance is not selected. In addition the authors do not compare their results with the clinical resistant mutations observed and tested on P. jirovecii's DHFR - Antimicrob Agents Chemother. 2013 Oct;57(10):4990-8. doi: 10.1128/AAC.01161-13.Epub 2013 Jul 29, nor the structural comparisons with active and remote mutations J Chem Inf Model. 2021 Jun 28;61(6):2537-2541. doi: 10.1021/acs.jcim.1c00403. Epub 2021 Jun 17.

2.2 Response: *It is true that the 2022 study in Antimicrob Agents Chemother does indeed not find a direct effect between intake of MTX and relief from P. jirovecii infection However, we believe that the conclusions from this study are limited by several factors. While several patients from this study had been diagnosed with P. jirovecii colonization, many had other lung conditions caused by different factors, such as some having bacterial pneumonia or iatrogenic lung disease, and all had other underlying conditions. As the outcome was simply stated as "improvement" or "deterioration", it is difficult to attribute individual patient outcomes to any one factor. Additionally, the authors of this study themselves acknowledge that their results could be affected by the relatively small size of the cohorts. Furthermore, another recent study has identified the opposing trend on the effects of MTX on the evolution of TMP resistance [2]. Despite this, we absolutely acknowledge that our study may not directly reflect clinical reality especially as the true impact of TMP in the TMP-SMX treatment of P. jirovecii is not well known and is debated upon, and the main text has been modified to reflect this more transparently. However, we still believe that studying mechanisms of resistance to other antifolates and working towards a better model to understand the evolution of resistance in this organism is a step in the right direction. The discussion has been modified to reflect this.*

Modifications in main text:

Lines 887-890 - Discussion: While this system does not allow the direct investigation of TMP resistance, we believe that in the light of the clinical and evolutionary link between MTX, TMP and *P. jirovecii* infection, this study provides relevant insights in the evolution of antifolate resistance, and on the biophysics of MTX resistance in DHFRs.

Lines 933 -938 - Discussion: However, as MTX is not used to treat *P. jirovecii* infections in clinical settings, and because of the conflicting reports of the effects of MTX on the evolution TMP resistance and on *P. jirovecii* infection, it is important to be critical about the nature of these results. We believe, however, that this is a step in the right direction, and that this data will help future studies focusing on more clinically relevant antifolates.

As for the study published in Antimicrob Agents Chemother. 2013 Oct;57(10):4990-8. doi: 10.1128/AAC.01161-13.Epub 2013 Jul 29, it is cited (see citation #80), and comparison with our own results is done for the main resistance mutation cited in the paper (F35D, see

lines 734 to 736), and all other mutations reported in this study are reported in S7_table.xlsx as other known resistance mutations in PjDHFR. The section discussing this paper has been expanded upon to include other key mutations of interest.

As for the study published in J Chem Inf Model. 2021 Jun 28;61(6):2537-2541. doi: 10.1021/acs.jcim.1c00403. Epub 2021 Jun 17, we indeed did not discuss the results and compare them to our own, but we did cite it (see citation #38). Specifically, this was omitted from the discussion in this paper, as the machine learning training dataset is the dataset from Antimicrob Agents Chemother. 2013 Oct;57(10):4990-8. doi: 10.1128/AAC.01161-13.Epub 2013 Jul 29, and therefore shows almost total overlap with this clinical dataset, and do not predict any new resistance mutations outside of what is reported in the paper from which they take their training dataset. Furthermore, the machine learning model was trained with a single dataset, which is limited to 20 mutations, and only showed a ~65% accuracy in its prediction when trying to predict the resistance effects of other mutants from this same dataset. Other factors limit the interpretability of these results. Firstly, the main predictor used in their model appears to be the amount of structural changes (RMSD) brought by the previously identified mutations, which the authors themselves mention cannot be correlated directly to resistance. Secondly, they omit to mention what percent of the variation does the principal components from their PCA analysis recapitulate, which heavily limits the interpretation from these results, as the weight of the components cannot be evaluated. However, we agree that we cannot disregard these results and predictions, and we expanded on the main findings from this study. The main text has been modified to include these comparisons.

Modifications in main text:

Lines 760-766 - Results: However, while some mechanisms are conserved, and cross resistance is observed, other sites do not share these properties. One such position is position L65, which was reported as conferring high resistance to TMP in clinical isolates, and no mutations at this site were identified as resistant in this screen [38]. Other mutations that were reported or predicted to confer TMP resistance, such as mutations in the Q172-V181 loop (compiled in S7 Table), did not confer resistance to MTX in this study [38].

2.3 The structural figure 4 does not show sufficient detail to understand how the resistance mutations even within the active site may be conferring resistance to MTX as opposed to the mutational scanning for function. At a quick glance the impact of mutations at F199 may be as simple as a repacking of the helix which packs against MTX.

2.3 Response: *Our study was primarily aimed at investigating mutations that confer resistance to MTX. The design of the screen in this particular study did not permit the direct testing of function at a high throughput. Conducting a systematic screening for function represents our next objective, and we are currently focusing our efforts on this.*

Our findings indicate that F35 could engage in stacking interactions with F199, suggesting that mutations at position F199 might influence the binding site in this manner. In this context, we posit that a distal mutation, which repacks a helix within the active site and induces resistance, can be considered an example of allostery, supporting our conclusions. The main text and Figure 4 have been updated to incorporate this new

analysis.

Modifications in main text:

Lines 820-822 - Results: Position F199 is also in close proximity to the side chain of position F35, and could be forming π-stacking interactions with this other phenylalanine, which could then affect the α-helix where position M33 is located [82].

Reviewer #3: In this manuscript, titled "Deep mutational scanning of Pneumocystis jirovecii dihydrofolate reductase reveals allosteric mechanism of resistance to an antifolate " by Rouleau FD, et. al., the authors explore methotrexate (MTX) resistance landscape of PjDHFR using deep mutational scanning (DMS) approach and pooled competition assay in yeast (S. cerevisiae). The authors derive selection coefficients for each substitution along the protein sequence by normalizing its log2-fold change in frequency between initial and final stages of growth in the absence and presence of two inhibitory concentrations of MTX (IC75 and IC90). Overall, 31 resistance-conferring positions along the protein sequence were discovered, for a total of 355 high-confidence resistance mutations. The authors then tested the validity of the obtained selection coefficients by engineering a fraction (44) of individual substitutions and testing their individual resistance effects. The authors concluded that most of the resistance-conferring positions along PjDHFR sequence identified in their study are located in close vicinity to the active site and coincide with the previously reported mutations in DHFRs of other origins. The analog of one antifolate-conferring substitution at position F199, however, was found only in bacterial and not eukaryotic DHFR sequences. Since F at position 199 is located far away from the active site and is buried within the hydrophobic core, the authors suggest an allosteric mechanism for substitutions at this position.

Major points.

3.1. The experimental system.

From the very beginning, the authors describe the development of a functional complementation assay to test the MTX resistance effects of mutations in PjDHFR. Specifically, in the Abstract, it is stated that " …by using functional complementation of the baker’s yeast ortholog…"; in the Introduction, "… We constructed yeast strains and plasmids that allow for the complementation of the Saccharomyces cerevisiae DHFR with PjDHFR". In the very first section of the Results, the authors introduce the design of the complementation system that is based on replacement of the yeast DHR1 gene with an MTX-resistance ortholog expressed from an inducible promoter and present the cartoon of the design as the first figure (Fig. 1a) in the manuscript. All this build up generates the ineluctable expectation that the presented functional complementation system will serve the basis for the mutational analysis. Particularly, because such a system could be used to test the effect of trimethoprim, the clinically relevant drug for P. jirovecii-caused pneumonia. However, this is not the case. The authors declare in as-a-matter-of-fact way that the system is ill suited for the high frequency transformation and, instead, a comprehensive scan of the mutational effects in PjDHFR is done on the background of functional DFR1 gene. This is a very misleading and confusing way of telling a story and should be avoided.

3.1 Response: *This is absolutely accurate. Experiments have been conducted to address this and the main text has been modified to make the story flow more comprehensively. Briefly, we expanded on: 1) How the results from both strains compare to each other, and demonstrate that the presence of genomic DFR1 has negligible effect on IC50, as well as the results from the screen. 2) Why we specifically decided to use strain IGA130 instead of FDR0001, as our goal was to test as many mutations as possible for resistance without introducing the bias for protein function in earlier transformation stages.*

We also conducted systematic growth curves in TMP for both strains, as a way to demonstrate how S. cerevisiae is insensitive to TMP in a DHFR-independent manner.

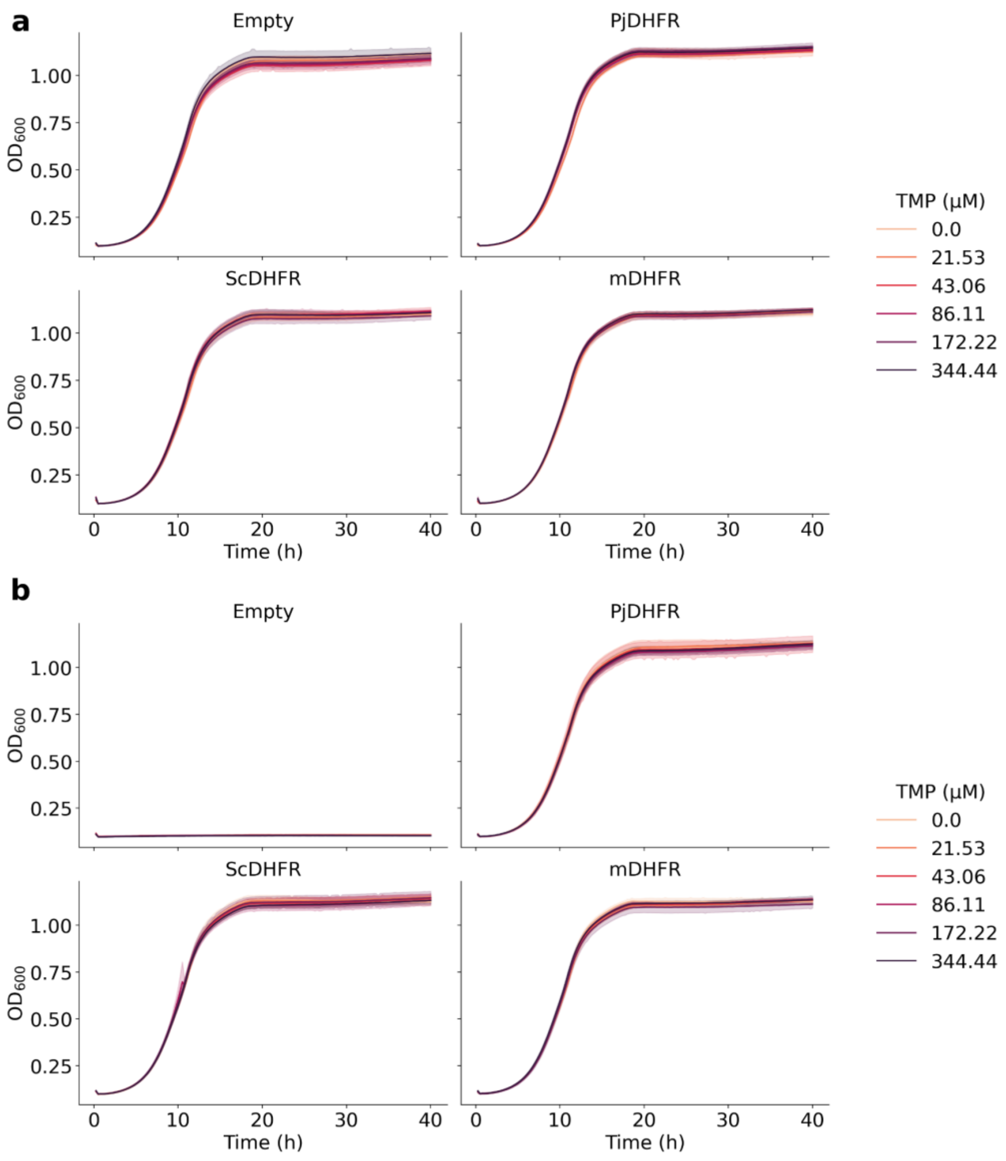

ReviewFig1: Dose response curve measurements for strain IGA130 and FDR0001 in the presence of TMP. a) *Growth curves of yeast strain IGA130 (wild-type for DFR1) each expressing a different DHFR. Shaded areas represent confidence intervals across biological triplicates. The strain appears to be insensitive to TMP regardless of the DHFR present. Measured IC50 for strain IGA130 with the Empty plasmid is ~17mg/mL, which is above the solubility limit of TMP in ethanol (~12mg/mL at RT) [1].* ***b)*** *Growth curves of yeast strain FDR0001 (dfr1Δ) each expressing a different DHFR. Shaded areas represent confidence intervals across biological triplicates. The strain appears to be insensitive to TMP regardless of the DHFR present.*

ReviewFig5, which shows individual growth curves for validation mutants in strain FDR0001, also shows the higher level of variance between replicates for mutants in strain FDR0001 compared to the same mutants in strain IGA130, illustrating some of the reproducibility and efficiency issues of working with strain FDR0001.

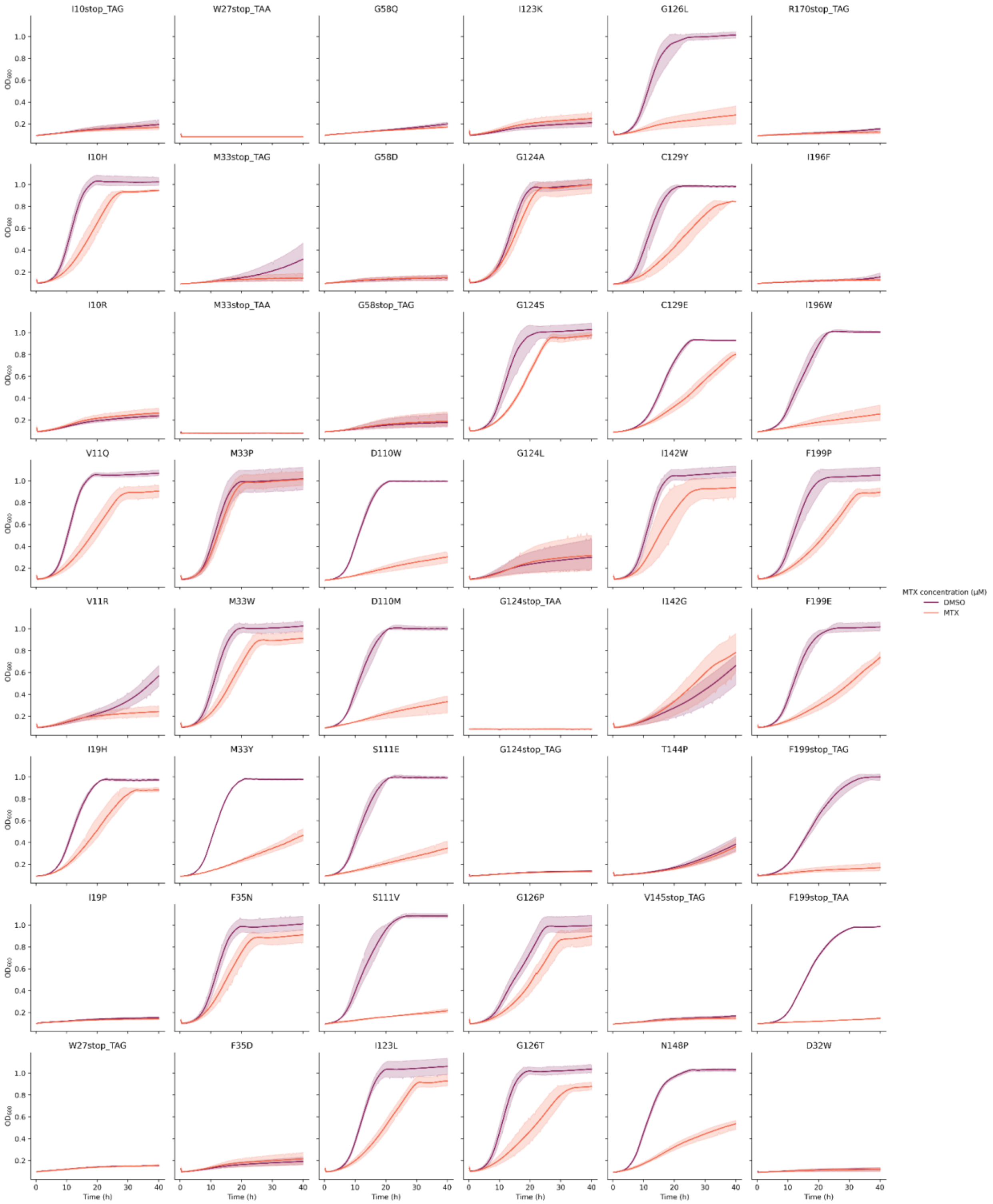

ReviewFig5: individual growth curves for validation of mutations in strain FDR0001 in DMSO and IC90. *Mutants are identified on top of their individual growth curves. For stop codons, we constructed both TAG (from DMS using NNK degenerate primers) and TAA as extra controls to ensure that TAG stop codons did not allow read through. All curves were done in triplicate, with error intervals between replicates appearing around the curve in a lighter color. Strains were grown in the same media as the competition assay for 40 hours, and validation growth rates were measured from these curves.*

Modifications in main text:

Lines 1149-152 - Introduction: We focus on the effects of mutations on MTX resistance. To ensure that we limit selection on mutations affecting protein function, we conduct the screening in a strain with a functional genomic DHFR, and then validate our results in a strain without a genomic DHFR to ensure the validity of our results.

3.2. Validation of the pooled competition experiment in FDR0001 (dfr1-) strain.

Out of the 44 individually engineered validation mutants only a fraction was tested in the FDR0001 strain. The identity of these mutants and why these particular mutants were chosen for the analysis is not specified anywhere. It is also not clear how their effects on growth and MTX resistance are correlated with those in the IGA130 strain (WT for FDR1). The comparison of the resistance trends between the strains is also not possible, since Fig. 3a that depicts behavior in the IGA30 strain presents correlations between selection coefficients at IC75 and IC90 MTX concentrations, whereas Fig S7a that relates to FDR0001 strain shows correlation between growth rates in DMSO and IC90 MTX. But it does look that some of the mutants with high selection coefficient (red dots in Fig S7a) grew poorly in the presence of IC90 MTX in the FDR0001, suggesting that there is a potential discrepancy between the mutational effects in IGA130 and FDR0001 strains. Since MTX is a competitive inhibitor of enzyme activity, presence of the background DHFR can mask the resistance profile of the identified mutations, particularly because addition of MTX can upregulate the expression of the endogenous DHFR. Therefore, it is important to validate the mutational effects in the absence of the background endogenous expression in the FDR0001 strain using all 44 engineered mutants and compare the obtained effect with those in IG130 strain.

3.2 Response: *We absolutely agree. This was a big oversight in the original version of the manuscript. Several experiments have been conducted to address this and modify the manuscript. Dose response curves have been conducted for strain FDR0001 to compare IC50 between it and IGA130, and all validation mutants have been transformed and validated in strain FDR0001. The measured growth rates between validations mutants in both strains have been correlated, and a high correlation has been identified between the two strains, highlighting the negligible effect of the genomic DFR1, and increasing confidence in the high-throughput screen results. As individual points are colored according to their selection coefficient from the DMS screening, some of the mutants appearing as red were identified as false positives when conducting validations. The main text has been modified to reflect this and figures have been added.*

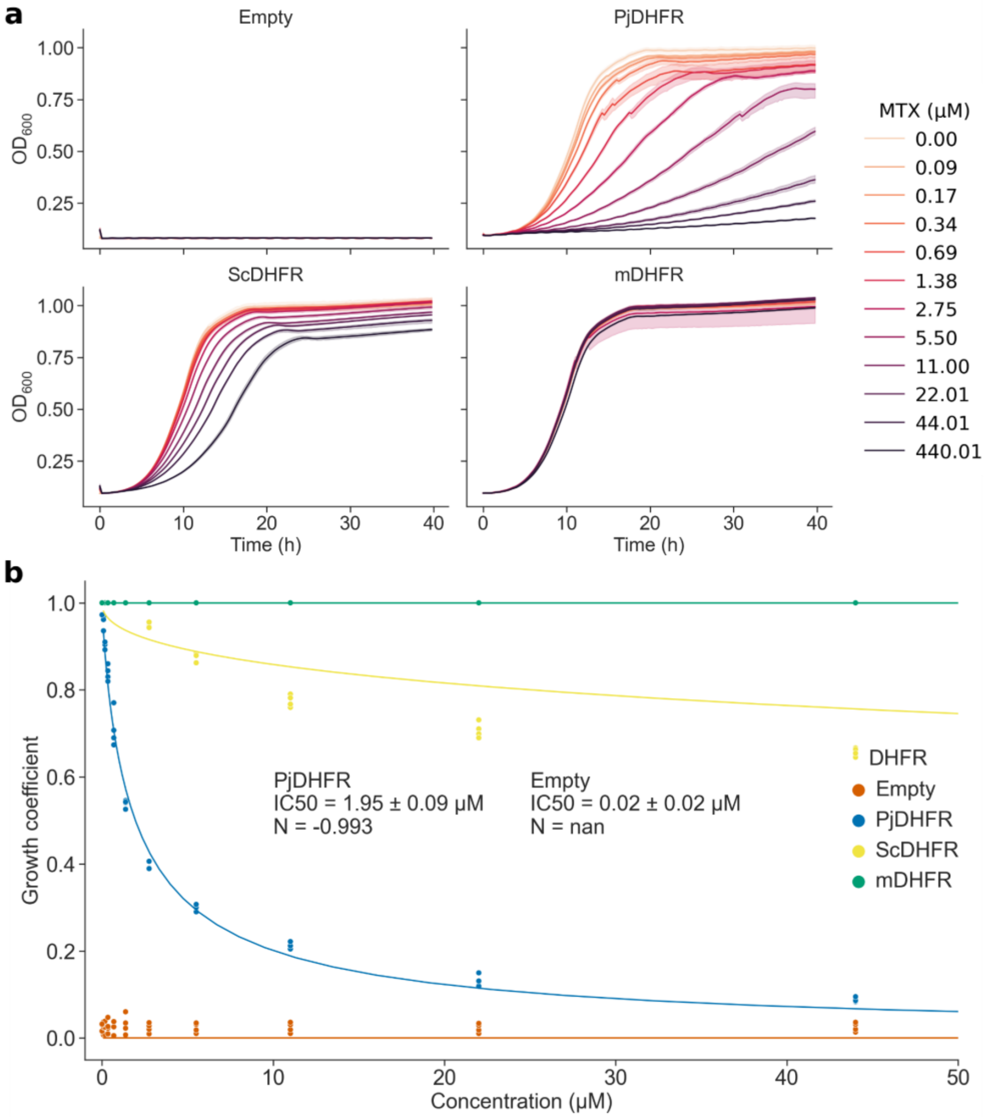

ReviewFig2: Dose response curve measurements for strain FDR0001 in the presence of MTX. **a)** Growth curves of yeast strain FDR0001 (null for *DFR1*) each expressing a different DHFR. Shaded areas represent confidence intervals across biological triplicates. The Empty vector does not support growth in this strain. Strains with vector-expressed DHFRs show increased resistance, with PjDHFR being the most sensitive, followed by ScDHFR, and mDHFR(L22F/F31S) being fully resistant. **b)** Growth coefficient (measure from growth rate relative to Empty without selection) of strain IGA130 with different DHFRs. R^2 for Hill equation fit: Empty = null, PjDHFR = 0.99, ScDHFR = 0.73 and mDHFR = >0.99.

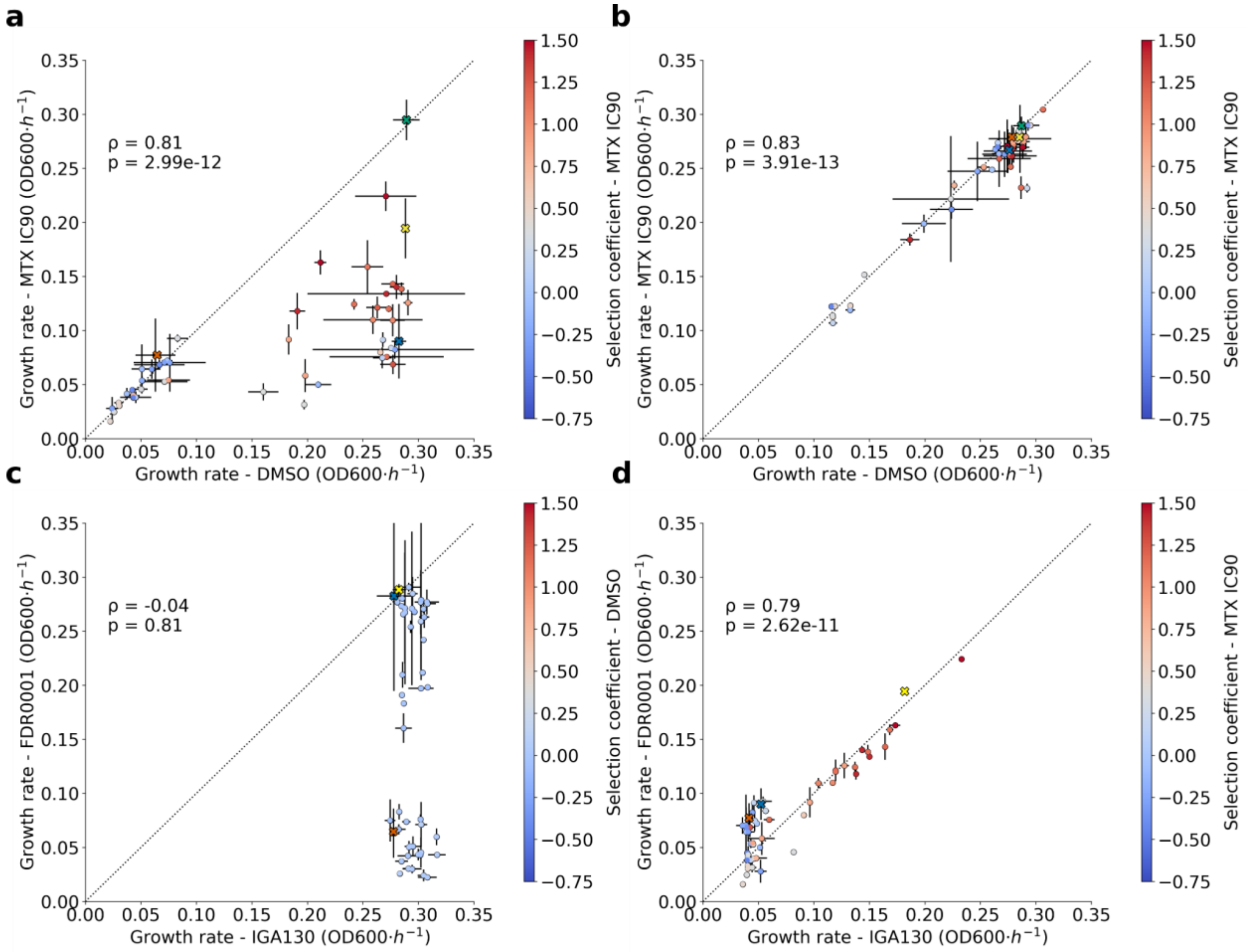

***ReviewFig3:*: Comparison of growth rates from validations growth curves in strain FDR0001 for some manually reconstructed mutants. a)** Growth rates in DMSO are plotted on the X axis, as a measure of complementation, and growth rates in the presence of MTX IC90 are plotted on the Y axis, as a measure of resistance. These growth curves were conducted in the absence of β-estradiol. Many sensitive mutants cannot complement the deletion of *DFR1* in strain FDR0001 (*dfr1Δ*). Error bars represent growth rate values measured across the biological replicates in both conditions. Data points marked by crosses represent the Empty plasmid (orange), the wild-type PjDHFR (blue), ScDHFR (yellow) and mDHFR (green). Other round data points are colored by their selection coefficients at IC90 unless specified otherwise. **b)** Same as panel a), but in the presence of β-estradiol, which leads to expression of MTX insensitive DfrB1. **c)** Comparison of growth rates between strains IGA130 (x-axis) and FDR0001 (y-axis) validation mutants in DMSO. All mutants can grow similarly in strain IGA130, but some mutants were non-functional in strain FDR0001. Color bar is the selection coefficient in DMSO. **d)** Comparison of growth rates between strains IGA130 (x-axis) and FDR0001 (y-axis) validation mutants in MTX IC90. For mutants that were identified as resistant, there is a strong correlation between growth rates measured in strain IGA130 and FDR0001, highlighting the minimal effect of the genomic DFR1 on MTX resistance. Non-functional mutants in FDR0001 had to be supplemented with 100nM β-estradiol to ensure growth of precultures. Statistical tests are Spearman's rank correlation.

<u>Main text modifications:</u>

Lines 555-561 - Results: To ensure that the use of strain IGA130 (wild-type for *DFR1*) did not introduce a bias in selection strength, we conducted identical dose response curves in strain FDR0001 (*dfr1Δ*) and estimated its IC50 as well (S3A, S3B Fig). IC50 measured for strain FDR0001 is of 1.95 µM, validating results from strain IGA130 on the

minimal effect of genomic DFR1 on MTX resistance. We believe that by using this system, we will be able to identify a maximum number of mutations in PjDHFR that confer resistance to MTX, without introducing downstream bias for PjDHFR activity.

Lines 667-672 - Results: We then used these results to correlate growth rate in IGA130 and in FDR0001 to validate how the results from the high-throughput assay could be transposed to strain FDR0001 (*dfr1Δ*). In DMSO, all mutants in strain IGA130 grew similarity, but non-functional PjDHFR mutants in strain FDR0001 were not able to grow. In the presence of MTX, the phenotype of resistant mutants are very well correlated between the two strains, highlighting the robustness of this assay (S7C and S7D Fig).

Lines 887-890 - Discussion: While this system does not allow the direct investigation of TMP resistance, we believe that in the light of the clinical and evolutionary link between MTX, TMP and *P. jirovecii* infection, this study provides relevant insights in the evolution of antifolate resistance, and on the biophysics of MTX resistance in DHFRs.

Lines 899-903 - Discussion: While individual validations in strain FDR0001 (*dfr1Δ*) allowed to identify resistance/function tradeoffs, the high-throughput screening in strain IGA130 (wild-type for DFR1) did not allow systematic investigation of this relationship. Further experiments will be necessary to understand the functional tradeoff associated with MTX resistance in PjDHFR.

3.3. The allosteric mechanism of resistance

The correlation in the drop of the predicted binding energy of the MTX-PjDHFR complex with the selection coefficients of mutants in position 199 is not statistically significant, and the trend is driven entirely by the two substitutions towards charged residues. But even if the correlation was statistically significant, I do not think that it is sufficient to substantiate the claim of allosteric mechanism of resistance, particularly emphasizing it in the Title as the major insight from the work. It is true that position F199 is not participating in binding to DHF/MTX and NADPH. But this position is deeply buried in the hydrophobic core. Thus, the most trivial explanation for the drop in MTX-PjDHFR complex binding energy is the destabilization of PjDHFR, which effectively lowers the frequency of productive encounters with MTX (but less so with DHF). In that case, the effect of substitutions at this position is not allosteric in the true sense of the word because there is no structurally-based interaction between the distant locations – active site and position 199. Another possibility for the MTX resistance found for the mutants in this position is their effect on DHFR abundance. If these mutations increase the DHFR abundance, e.g., through dimerization of the destabilized monomers, the MTX resistance would rise. In this case, again, the mutational mechanism is not allosteric.

3.3 Response: *Indeed, some mutations could cause increased protein stability, and therefore abundance, which could lead to increased resistance. This is a well documented mechanism of resistance in DHFRs [8]. To investigate this possibility, we constructed PjDHFR-mEGFP fusions using some of the validation mutants, and measured fluorescence intensity using flow cytometry as a proxy for protein abundance (5000 events, in triplicate). We did not observe a correlation between resistance and protein abundance (see ReviewFig5A and B). Mutants I10H and I10R (selection coefficients of 1.154 and 0.014, respectively) both had*

reduced abundance (0.71 and 0.82 fold change in relative fluorescence intensity per cell, respectively.) As for position 199 specifically, we had only reconstructed two validation mutants (F199P and F199E, selection coefficients of 1.018 and 0.585, respectively, both resistant, since no mutations at position F199 leads to a negative selection coefficient.) These two mutants showed slightly increased expression levels, but the measured increase in expression is not proportional with the measured selection coefficient (F199P and F199E, with increase of 1.05 and 1.02 fold-change in relative fluorescence intensity per cell, respectively). Based on these observations, we believe that MTX resistance cannot be solely attributed to increase in protein abundance, but it is possible that it is a contributing factor. The main text has been modified to reflect this.

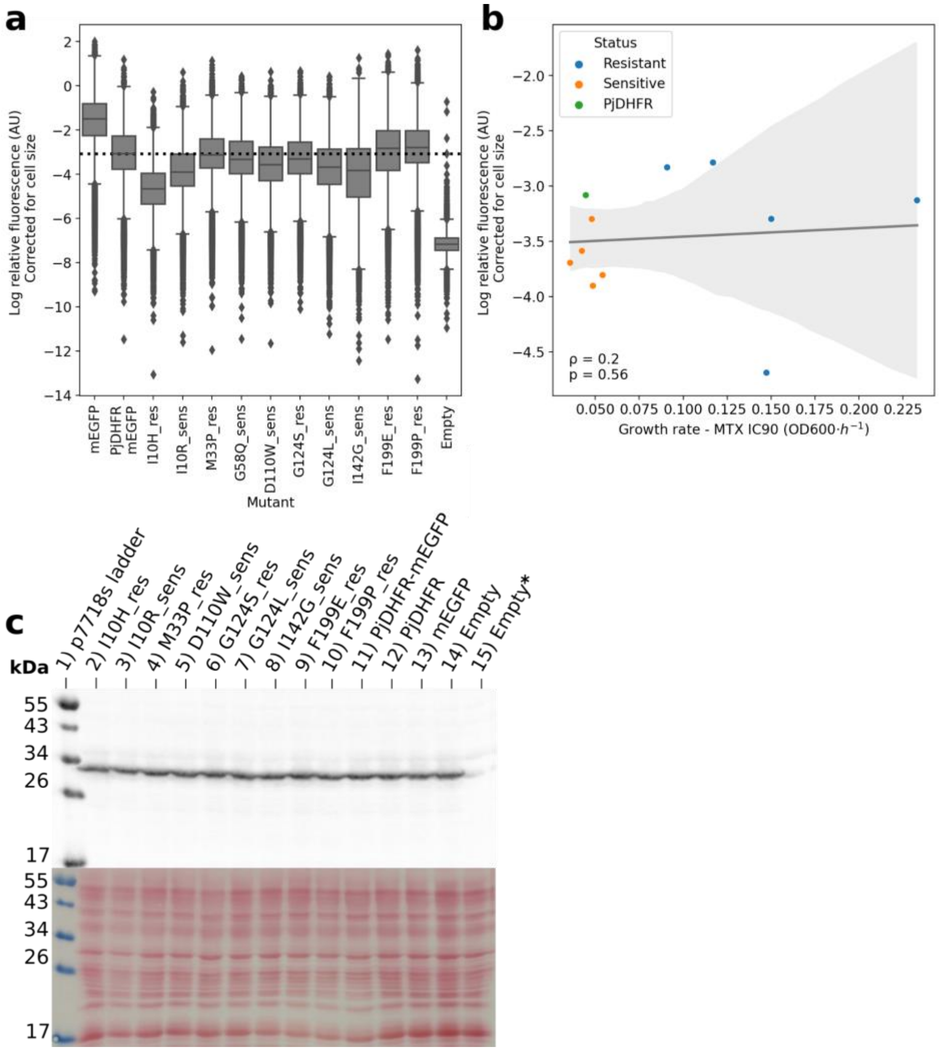

ReviewFig4: Measurements of fluorescence intensity of PjDHFR mutants tagged with mEGFP as a proxy for protein abundance. **a)** Flow cytometry measurements for different validation mutants and controls in strain IGA130. mEGFP represents mEGFP alone expressed under the regulation of the same promoter used for the screen. PjDHFR-mEGFP is the wild-type PjDHFR tagged with mEGFP. Mutants marked with sens or res are mutants that are identified as sensitive or resistant, respectively. Empty is the empty plasmid as a negative control. **b)** Correlation between median measured fluorescence and growth rate in MTX IC90, collared by mutant status. No correlation was found between expression levels and resistance. **c)** Anti-FLAG western blot and Ponceau S red stains of strain FDR0003 (DFR1 3xFLAG-tag) to measure changes in expression levels of genomic DFR1 when co-expressed with PjDHFR mutants. 11) is

PjDHFR-mEGFP fusion protein, 12) PjDHFR is PjDHFR wild-type without fusion, 13) is mEGFP without fusion, and 14) is Empty plasmid. All transformations were done in strain FDR0003 unless specified. 15) Empty***** is Empty plasmid transformed in strain IGA130 as a FLAG-negative control.

This figure is now figure S12 in the revised manuscript.

After further discussion with collaborators and structural analysis, we have indeed concluded that position F35 could form stacking interactions with position F199, and therefore that mutations at position F199 could affect the binding site in this way. However, we consider that a distal mutation repacking an helix in the active site and causing resistance to still be allostery, we believe that our conclusions still hold as it does not involve a direct contact between the site and the drug. The main text has been modified to reflect these new analyses and figure 4 has been modified to reflect this as well.

Furthermore, we expanded on the statistical analyses of the correlations between changes in binding affinity of mutations at position F199 with selection coefficient. As it is not possible to increase the number of datapoints on this graph with either experiments or modeling, we decided to test for different levels of correlation. When considering Pearons correlation coefficient, a linear correlation with statistical significance has been established, highlighting covariance between those two datasets. Figure 4 has been modified to reflect this. The relationship between DMS results and flexddG modeling will be expanded upon in future works.

<u>Modifications in main text about alternative mechanisms of resistance:</u>

Lines 820-822 - Results: Position F199 is also in close proximity to the side chain of position F35, and could be forming π-stacking interactions with this other phenylalanine, which could then affect the α-helix where position M33 is located [81].

Lines 850-877 - Results: **Alternative mechanisms of MTX resistance in PjDHFR**

Alternative mechanisms of resistance were also investigated. Indeed, antifolate resistance caused by increased DHFR expression or protein abundance has been documented. To verify this, we constructed PjDHFR-mEGFP fusion proteins for wild-type PjDHFR and some validation mutants of interest. Fluorescence intensity was measured by flow cytometry as a proxy for protein abundance (S12A Fig.). As all mutants are under the regulation of the same promoter, variation in abundance should depend on changes in protein stability caused by the mutations. We did not observe a correlation between resistance and protein abundance (S12B Fig.). Mutants I10H and I10R (selection coefficients of 1.154 and 0.014, respectively) both had reduced abundance (0.71 and 0.82 fold change in relative fluorescence intensity per cell, respectively), where the most resistant mutant of the two is the least abundant. As for position 199 specifically, we had only reconstructed two validation mutants (F199P and F199E, selection coefficients of 1.018 and 0.585, respectively, both resistant, since no mutations at position F199 leads to a negative selection coefficient). These two mutants showed slightly increased expression levels, but the measured increase in expression is not proportional with the measured selection coefficient (F199P and F199E, with increase of 1.05 and 1.02 fold-change in relative fluorescence intensity per cell, respectively). Based on these observations, we

believe that MTX resistance cannot be solely attributed to increase in protein abundance, but it is possible that it is a contributing factor.

As the screen was conducted in strain IGA130 (wild-type for genomic DFR1), it was also possible that the expression of PjDHFR mutants from the plasmid caused fluctuations in the expression of genomic DFR1, affecting measured DMS scores. To test this, we constructed strain FDR0003, which is the same as strain IGA130, but where genomic DFR1 is tagged with 3xFLAG-tag in C-terminal. We measured changes in genomic DFR1 expression by anti-FLAG western blot in the presence of several resistant and sensitive mutants, and did not observe any changes in DFR1 expression in the presence of PjDHFR mutants (S12C Fig.)

Lines 950-953 - Discussion: While protein abundance measurements showed no clear correlation with resistance in this study, increase in protein stability has been well documented as an antifolate resistance mechanism, and at least two of the tested resistant mutants showed increase in protein abundance.

Have all data underlying the figures and results presented in the manuscript been provided?
Large-scale datasets should be made available via a public repository as described in the *PLOS Genetics* data availability policy, and numerical data that underlies graphs or summary statistics should be provided in spreadsheet form as supporting information.

Reviewer #1: Yes

Reviewer #2: Yes

Reviewer #3: **No:** Please see Major point 2

PLOS authors have the option to publish the peer review history of their article (what does this mean?). If published, this will include your full peer review and any attached files.

If you choose "no", your identity will remain anonymous but your review may still be made public.

Do you want your identity to be public for this peer review? For information about this choice, including consent withdrawal, please see our Privacy Policy.

Reviewer #1: No

Reviewer #2: No

Reviewer #3: No

References

1. Zhang N, Li S, Yang H, Li M, Yang Y, Tang W. Measurement and Correlation of the Solubility of Tetramethylpyrazine in Nine Monosolvents and Two Binary Solvent Systems. J Chem Eng Data. 2019;64: 995–1006.

2. Guðmundsdóttir JS, Fredheim EGA, Koumans CIM, Hegstad J, Tang P-C, Andersson DI, et al. The chemotherapeutic drug methotrexate selects for antibiotic resistance. EBioMedicine. 2021;74: 103742.

3. Mori S, Sugimoto M. Pneumocystis jirovecii infection: an emerging threat to patients with rheumatoid arthritis. Rheumatology . 2012;51: 2120–2130.

4. Ohmura S-I, Homma Y, Masui T, Miyamoto T. Factors Associated with Pneumocystis jirovecii Pneumonia in Patients with Rheumatoid Arthritis Receiving Methotrexate: A Single-center Retrospective Study. Intern Med. 2022;61: 997–1006.

5. DeJarnette C, Luna-Tapia A, Estredge LR, Palmer GE. Dihydrofolate Reductase Is a Valid Target for Antifungal Development in the Human Pathogen Candida albicans. mSphere. 2020;5. doi:10.1128/msphere.00374-20

6. Dornfeld K. Antifolate response in replication arrest mutants of Saccharomyces cerevisiae. Anticancer Res. 2013;33: 2037–2041.

7. Bilsland E, Pir P, Gutteridge A, Johns A, King RD, Oliver SG. Functional expression of parasite drug targets and their human orthologs in yeast. PLoS Negl Trop Dis. 2011;5: e1320.

8. Visentin M, Zhao R, Goldman ID. The Antifolates. Hematol Oncol Clin North Am. 2012;26: 629–629.
